# State of science: occupational slips, trips and falls on the same level^*^

**DOI:** 10.1080/00140139.2016.1157214

**Published:** 2016-03-30

**Authors:** Wen-Ruey Chang, Sylvie Leclercq, Thurmon E. Lockhart, Roger Haslam

**Affiliations:** ^a^ Liberty Mutual Research Institute for Safety, Hopkinton, MA, USA; ^b^ French National Research and Safety Institute (INRS), Vandoeuvre, France; ^c^ School of Biological and Health Systems Engineering, Ira A. Fulton Schools of Engineering, Arizona State University, Tempe, AZ, USA; ^d^ Loughborough Design School, Loughborough University, Loughborough, UK

**Keywords:** Slips, trips and falls, workplace falls, fall causation, fall prevention, occupational injury prevention

## Abstract

Occupational slips, trips and falls on the same level (STFL) result in substantial injuries worldwide. This paper summarises the state of science regarding STFL, outlining relevant aspects of epidemiology, biomechanics, psychophysics, tribology, organisational influences and injury prevention. This review reaffirms that STFL remain a major cause of workplace injury and STFL prevention is a complex problem, requiring multi-disciplinary, multi-faceted approaches. Despite progress in recent decades in understanding the mechanisms involved in STFL, especially slipping, research leading to evidence-based prevention practices remains insufficient, given the problem scale. It is concluded that there is a pressing need to develop better fall prevention strategies using systems approaches conceptualising and addressing the factors involved in STFL, with considerations of the full range of factors and their interactions. There is also an urgent need for field trials of various fall prevention strategies to assess the effectiveness of different intervention components and their interactions.

**Practitioner Summary**: Work-related slipping, tripping and falls on the same level are a major source of occupational injury. The causes are broadly understood, although more attention is needed from a systems perspective. Research has shown preventative action to be effective, but further studies are required to understand which aspects are most beneficial.

## 1. Introduction

Occupational slips, trips and falls on the same level (STFL) are a serious problem, with substantial injury and economic consequences reported worldwide. Although the scale of the problem has been recognised over several decades (Strandberg and Lanshammar 1981; Buck and Colman 1985; Leamon and Murphy 1995; Kemmlert and Lundholm 1998; European Agency for Safety and Health at Work (EU-OSHA) 2001; European Commission 2008; Nenonen 2013; Yeoh, Lockart, and Wu 2013), STFL persist as a major source of workplace injuries. The most recent data from the Liberty Mutual Workplace Safety Index, for example, showed that the direct cost of disabling workplace injuries in 2012 due to falls on the same level in the US was estimated to be approximately US$9.19 billion or 15.4% of total injury cost (Liberty Mutual Research Institute for Safety 2014).

STFL have received the concerted attention of safety researchers, with progress made in understanding their mechanisms and circumstances. The complexity of the interacting causal factors, however, intrinsic and extrinsic, close to or upstream from the injury genesis, presents a considerable challenge in designing and implementing effective prevention strategies. This state of science article first considers relevant definitions and establishes the scope of the review, proceeding to describe occupational STFL from different disciplinary perspectives. This consists of a discussion of the factors involved, examining epidemiological, biomechanical, perceptual (i.e. psychophysical), tribological and organisational aspects. The review concludes by summarising the current state of knowledge regarding STFL prevention and the areas where further research is required. The review is structured to reflect the critical research approaches that have been brought to bear on the STFL problem over the past three decades.

## 2. Definitions and scope

Falls on the same level, on stairs and from heights are endemic throughout society, afflicting all ages. Likewise, falls occur widely during home and leisure pursuits as well as those related to work. The scope of this paper, however, is limited to occupational STFL as a particular class of injuries. The distinction between occupational and non-occupational slips, trips and falls, and falls on the same level vs. falls on steps and stairs or from heights, is significant when incidents are considered from a systems perspective. Different causal factors are dominant in occupational slips, trips and falls compared with falls among the elderly, for example, with different patterns of causation leading to different approaches to prevention. Further justification for limiting the scope of this review to occupational STFL is that the distinction also reflects the practice of different communities of researchers, practitioners and their disciplinary backgrounds.

Early research in the field used the expression ‘slipping, tripping and falling accidents (STFA)’ which, as far as we are aware, was first introduced in the early 1980s at a dedicated international conference held in the United Kingdom (Davis 1983a). Later, the concatenated ‘slips, trips and falls (STF)’ entered widespread use (e.g. Haslam and Stubbs 2006; Chang 2008), embracing falls on the same level, falls from height and falls from some other action (e.g. movement of the standing surface). It is notable that slipping has been a particular area of attention, forming a large part of the discussions at STF symposia over the years, for example, at the Liberty Mutual Research Institute for Safety international symposium on the measurement of slipperiness (Courtney et al. 2001a). Some researchers have, however, suggested that use of the ‘slips, trips and falls’ terminology and the tendency to focus on slipping fail to give sufficient recognition to other important causes of injuries on level surfaces involving loss of balance (Lortie and Rizzo 1999) or movement disturbance (Leclercq, Monteau, and Cuny 2010; Leclercq et al. 2014; Leclercq et al. 2015).

Slipping occurs when the friction between the foot or shoe sole and the floor surface provides insufficient resistance to counteract the forward or rearward forces that occur during the stepping process, i.e. interaction between human (foot or shoe sole) and floor. Leamon and Li (1990) described three categories of slips when walking based on the length of a slip. A ‘microslip’ is a slip shorter than 3 cm, a ‘slip’ is as long as 8–10 cm, and a ‘slide’ describes uncontrolled movement of the heel, which typically arises when a slip length exceeds approximately 10 cm. Microslips generally pass unnoticed; a slip will result in instinctive efforts to regain postural control; a slide is likely to lead to a loss of balance resulting in a fall. A trip occurs when the swing phase of the foot is interrupted unexpectedly due to inadequately clearing the ground. Irregularities of as little as 5 mm in the walking surface may be sufficient to cause a trip (Begg, Best, and Taylor 2007). Loss of balance leading to falls can also arise due to unexpected, forcible contact with something or someone. Similarly, unexpected, forcible movement of the floor, as may happen when standing in a moving vehicle, may cause a loss of balance sufficient to result in a fall on the same level.

The notion of ‘stumbling’ has been present in commentaries on falls and accident classifications (e.g. Strandberg 1983; European Commission 2013). The European Commission (2013) has ‘injured person slips, stumbles or falls on the same level’ as one of the deviation codes in the European Statistics on Accidents at Work. In this context, however, ‘stumbles’ is not defined and appears to be used in place of ‘trips’, which does not appear in the classification scheme. For the purposes of this review, it is assumed that stumbling is a consequence of slipping, tripping or other loss of balance event and refers to the process of falling and subsequent attempts to regain balance, rather than being a triggering event for a fall incident.

Situations referred to as ‘stepping into air’ when walking and missing a low, unmarked step down, for example, are regarded for the purposes of this paper as falls on steps or stairs and beyond the scope of the current focus on STFL. Likewise a fall arising from slipping on the lower rungs of a ladder or a slip or trip on scaffolding leading to a fall from height is excluded. Slips, trips and falls when walking on a slope (ramp, hill etc.) involve a change of height and have different biomechanics, tribology and loss of balance characteristics to STFL, so they are not covered. Falls arising from the collapse of an individual due to intrinsic factors, such as a health condition, are also not considered in the present review. Although patient and older person falls in hospitals or other health or social care environments can occur in a workplace setting, these are omitted from this review as a category of falls in their own right, with a distinct pattern of causal factors and a distinct body of research to match (e.g. Cameron et al. 2012).

## 3. Epidemiology

Examining patterns of occurrence of STFL aids understanding of the distribution of STFL risks across different industries, occupations and worker groups. Firstly, however, it is appropriate to comment on limitations with the available data. Such data, whether collected as part of national, industrial sector or company occupational injury monitoring schemes, rely on classification systems that vary and are necessarily restricted in their categories. Strandberg (1983) highlighted the distortion of data that can arise with reporting schemes requiring classification of an incident into a restricted number of groupings, compounded further by coding choices having to be made by registration personnel based on their subjective judgements. Strandberg’s analysis found that slipping and falling incidents to have been seriously underestimated with the reporting scheme applied in Sweden at the time.

Some incidence reporting schemes may fail to differentiate between falls on the same level and falls from height (Lortie and Rizzo 1999). With the most recent occupational injury data available in Great Britain, for example, ‘slips, trips and falls on the same level’ and ‘falls from height’ are presented and discussed together as ‘slips, trips and falls’, as the information collected does not allow a consistent distinction to be made between them (Health and Safety Executive 2014). Another point to note is that variation exists in the precise classifications used by different reporting schemes for incidence data relevant to STFL. The variation in the terminology in the following sections reflects that used by different reporting agencies and researchers.

### 3.1. Scale of STFL problem

The European Commission (2008) presented an analysis of 3,983,881 non-fatal accidents at work occurring during 2005, involving more than 3 work days absence. Of these, ‘slipping – stumbling and falling – fall of person – on the same level’ was the largest category, amounting to 14.4%. A further 4.4% were recorded as ‘treading badly, twisting leg or ankle, slipping without falling’. In the US, data from the Bureau of Labor Statistics (BLS) (2014) showed that among 1,162,210 non-fatal occupational accidents and diseases recorded in 2013 at private companies and government agencies, 17.4% were a fall on the same level resulting in a median 10 work days lost. A further 4.4% of the reports were slips or trips without a fall but leading to an injury (e.g. back injury), resulting in a median 11 work days lost. Thus, two important data collection agencies indicate that STFL internationally amount to approximately 1 in 5 of reported non-fatal work-related accidents. Reliable occupational injury data are only available for a limited number of countries, but the data that do exist indicate that occupational injury rates are much greater in countries beyond those classified as ‘established market economies’ (Hämäläinen, Takala, and Saarela 2006). Even with the caveats that apply in extrapolating data available from industrially developed countries, we can be confident that the global toll of injury from STFL is immense.

In industrially developed countries, the overall number of work-related injuries has shown a decline, whereas injuries from STFL as a proportion have increased. Examination of occupational accidents with work days lost at companies operating within the French general social security system, for example, revealed an overall reduction of 13.6 accidents/1000 employees between 1987 and 2011 (CNAMTS 1988, 2012). For injuries triggered by a slip, trip or any other movement disturbance, excluding falls from height, however, the reduction was only 1 accident/1000 employees. Similarly, analysis of Liberty Mutual Workplace Safety Index data revealed that the cost of falls on the same level increased by 42.3% between 1998 and 2010, after adjusting for inflation, while the overall costs of disabling workplace injuries decreased 4.7% over the same period (Liberty Mutual Research Institute for Safety 2012). There is a broad indication that, although there has been much success with wider workplace injury prevention, the prevention actions have not addressed STFL as effectively as other accidents at work.

### 3.2. Sectorial variations

The incidence of STFL varies with the nature of work, the context in which it is undertaken and consequent variation in exposure to STFL hazards. An early analysis by Buck and Coleman (1985) showed that the frequency rate for STFL per 10,000 employees in 30 industrial sectors varied between 227 in mining and quarrying and 4 in banking and insurance. Significant variations were also observed in the groups within each industrial sector. Leclercq and Thouy (2004) and Leclercq, Thouy, and Rossignol (2007) showed that employees were differently affected by STFL leading to work days lost, depending on their occupation and even on the type of equipment with which they worked: rail ticket inspectors were differently affected (by a factor of 1 to 10), depending on the type of train in which they were operating. Gaudez, Leclercq, and Derosier (2006), in an analysis of 2002 data for companies operating within the French general social security system, found that in 9 industrial sectors, the rates relating to STFL leading to days lost were highest in the building and civil engineering sectors. In comparison, rates were approximately 5 times lower in service sectors. Analysis of US BLS data for 2011 by Yeoh, Lockart, and Wu (2013) revealed that workers with an occupation of ‘healthcare support’ and ‘transportation and material moving’ were the most affected by same level falls leading to one or more days away from work at 40.6 accidents and 31.6 per 10,000 workers, respectively. On the contrary, employees in the office and administration industrial sector were the least affected at 10.2 accidents per 10,000 workers.

### 3.3. Age, gender and obesity

Several studies have investigated the relationship between age and occupational STFL; the findings reported in the literature are sometimes contradictory. Buck and Coleman (1985) found that the frequency rate for STFL increased with employees’ age (between 16 and 60 years). Yeoh, Lockart, and Wu (2013) found a similar trend of incidence increasing with age, but with a slightly higher incidence rate in the youngest workers (between 16 and 19 years old) than in the workers aged between 20 and 44 years old. Kemmlert and Lundholm (1998) observed that workers older than 45 years were more often victims of slips, trips and falls than younger workers.

On the other hand, at a more detailed level examining company records for a particular occupation, mail delivery workers, Bentley and Haslam (1998) did not find any significant relationship between age and the occurrence of slip, trip and fall accidents. Similarly, research reported by Kong, Suyama, and Hostler (2013) for US and Polish firefighters found slips, trips and falls not to be associated with age. At three of four companies studied by Leclercq and Thouy (2004) and Leclercq, Thouy, and Rossignol (2007), younger employees experienced more STFL than their older counterparts. In a prospective investigation of slips among workers in fast food restaurants, Courtney et al. (2013) found that slipping occurrence decreased with increased age. In addition to being at variance with the pattern observed in the larger incidence data-sets above, these contradictory findings might be considered unexpected in view of the literature dealing with the association between age, health, physical condition and balance. This literature, however, mostly addresses falls among older people (65+) in the non-working population (e.g. Pyykkö, Jantti, and Aalto 1990; Alexander et al. 1992; Perrin and Lestienne 1994). The study of Bentley and Haslam (1998) may indicate that increased susceptibility to STFL with ageing only becomes a factor in older workers who have had a certain level of age-related changes regarding fitness, strength and balance. It is also possible that some types of work may require a certain level of fitness, beneficial in alleviating susceptibility to STFL regardless of age. This may be coupled with individuals not attaining or maintaining this level of fitness moving out of the occupation. Another possibility could be that the difference with age in some work situations is accounted for by influences such as experience, environmental familiarity, reduced risk taking and the nature of work tasks allocated (Leclercq and Thouy 2004) and Leclercq, Thouy, and Rossignol (2007).

With regard to gender differences in incidence, Yeoh, Lockart, and Wu (2013) analysis of US BLS 2010 data found female workers had a higher rate of same level falls leading to one or more days away from work than male workers (21.7 vs. 12.1 per 10,000 workers). Examining European accident data, Nenonen (2013) found that when compared with other accidents at work, the proportions of female workers experiencing slipping, stumbling or falling was higher. The pattern with gender differences when considering particular occupations is less clear. Bentley and Haslam (1998) found an incidence rate among mail delivery workers approximately 50% greater among females than their male colleagues (12.8 vs. 8.2 per 10,000 workers). Courtney et al. (2013), however, found no difference between male and female workers in a prospective study of slips in fast food restaurants. One explanation for possible gender differences in STFL incidence is the gender variation in the composition of the workforce for different occupations and corresponding variation in exposure to STFL risk. Another reason might relate to differences in stature and strength, with females operating at a greater percentage of their capacity for more strenuous tasks. Indeed in the study by Bentley and Haslam (1998), the postal delivery task involved carrying a heavy asymmetric load (mail pouch). It is possible this acted as a greater encumbrance for female workers, having a greater effect on balance and ability to recover balance in the event of an STFL initiating event.

Another physical attribute that appears to have an influence on STFL is body mass. Being overweight was found to be related to falls on the same level among male construction workers (Chau et al. 2004). Similarly, Koepp, Snedden, and Levine (2015) found obesity to be related to slip, trip and fall injuries among workers at an applied engineering facility. A recent gait experiment involving young obese adults suggests that slip-induced fall risks are higher along the transversal direction due to wider step width (Wu, Lockhart, and Yeoh 2012). Miller, Matrangola, and Madigan (2011), however, found no differences in balance recovery from small externally applied perturbations between obese and normal participants.

### 3.4. Injury outcomes

Fortunately, fatalities are a rare consequence of STFL. Nevertheless, Buck and Coleman (1985) emphasised that injuries from STFL are far from trivial, with 17% of those in their examination of published data on workplace accidents in Great Britain resulting in fractures. A further 17% were classified as ‘contusions and crushing’ and 36% as ‘sprains and strains’. Bentley and Haslam (1998), in a study of postal delivery workers working outdoors, found that the ankle was the most frequent site of injuries (23%), followed by the knee (17%) and back (16%). They also found that almost 50% of days lost were due to ankle and back problems; ankle injuries resulted most often from trips and back injuries from slips. For US workers experiencing injuries requiring days away from work, Yeoh, Lockart, and Wu (2013) found that extremities, which included the knees, feet and toes, were the most affected body parts injured in falls on the same level, comprising 30.7%. The trunk, which included shoulder and back, was the second most injured at 25.6%. Workers with multiple injured body parts ranked third with 21.8% of overall injuries.

In the industrial environment, back injury is the most frequent cause of workers’ compensation claims in the United States (Guo et al. 1999). The prevalence of low-back pain in a life-time has been reported to be between 55% and 87% (Videman et al. 1984; Riihimäki 1985). Low-back pain has been shown to be associated with slips and falls (Rohrlich et al. 2014). Epidemiological studies have indicated that sudden loading to the trunk is associated with acute low-back pain and may be a primary risk factor for chronic low-back pain development (Manning and Shannon 1981; Manning, Mitchell, and Blanchfield 1984; Rohrlich et al. 2014). Courtney et al. (2001b) suggested that one workers’ compensation provider claimed that the cost ratio for ruptured discs due to same level falls was highest (13.3) among many injury claims (*cost ratio* is the ratio of the average cost of the particular injury to the average cost of all injuries for that particular class of falls). Unexpected gait perturbations can be dangerous to the lumbar spine because of the rapid corrective movements needed to regain balance (Liu, Lockhart, and Kim 2014). Trunk acceleration can increase significantly during unexpected perturbation, such as slipping, compared with that during normal gait (Hirvonen et al. 1994; Ehsan et al. 2013).

## 4. Biomechanics

This section describes some individual and task factors that may influence fall occurrence and severity. The knowledge generated based on biomechanics could complement that from existing injury surveillance systems. Understanding these factors contributes towards the development of fall prevention strategies. The examination also reveals where gaps in knowledge exist and further research is required.

Human bipedal locomotion (walking) is a challenging function for the central nervous system (CNS). During the single support period, which accounts for 80% of a gait cycle, the body is in a continuous state of instability since the whole body’s centre-of-mass is outside of the base of support (i.e. foot edge) (Perry 1992). The dynamic stability is recovered after the swing limb establishes firm contact with the ground. As such, dynamic stability is lost and regained in every gait cycle during normal walking. This recovery requires a complex interplay of neural and motor control mechanisms. Motor control is directly linked to the CNS’s processing of sensory inputs (visual, vestibular and proprioceptive). The sensory systems send input to an instantaneous controller to make an adjustment in real time. Additionally, an internal model is used to predict and adapt into the next step. It is clear that ‘expectancy’ is valuable for safe walking (Sicre et al. 2008). There can be a motion perturbation such as a slip, trip or a loss of balance if expectation and reality do not match. If not controlled, this perturbation could develop to become a fall.

### 4.1. Slips when walking

Falls initiated by slips are the most prevalent STFL (Courtney et al. 2001b) and have received concerted research attention. A slip occurs at the shoe and floor interface when the friction required (required coefficient of friction, RCOF) to support walking exceeds the friction available (available coefficient of friction, ACOF) at the shoe and floor interface (Hanson, Redfern, and Mazumdar 1999). The RCOF for straight walking has been investigated extensively (Hanson, Redfern, and Mazumdar 1999; Cham and Redfern 2002a; Kim, Lockhart, and Yoon 2005; Chang, Matz, and Chang 2012; Anderson, Franck, and Madigan 2014; Beringer, Nussbaum, and Madigan 2014; Fino and Lockhart 2014; Fino, Lockhart, and Fino 2015). The transverse component of the ground reaction force obtained with a force plate has been ignored in most of the RCOF calculations, but a study conducted by Chang, Chang, and Matz (2011) demonstrated that the transverse shear force could significantly increase RCOF and result in a much earlier occurrence of RCOF in the gait cycle. Recent studies showed that the RCOF for turning could be as high as 0.36, while that for straight walking was of the order of 0.2 (Burnfield, Tsai, and Powers 2005; Yamaguchi et al. 2013; Fino, Lockhart, and Fino 2015). The RCOF for carrying out different tasks under different situations, such as walking on different floor surfaces with different footwear, needs to be investigated further.

Much of the literature on biomechanical aspects of slips is concerned with human responses to unexpected contamination on floor surfaces (Lockhart 1997; Cham, Beschorner, and Redfern 2007; Lockhart, Woldstad, and Smith 2003; Lockhart, Smith, and Woldstad 2005; Lockhart 2008). Some of the research focus has been on the kinematic measurements at heel contact immediately before a slip incident and the body responses to a slip event. Parameters measured included heel displacements and velocities, joint angles and body part positions. For the kinematics immediately before a slip event, research has focused on identifying parameters associated with RCOF measurement or slip outcomes and anticipated reactions to potentially slippery floor surfaces (Kim, Lockhart, and Yoon 2005; Moyer et al. 2006; Hu and Qu 2013). Elsewhere, velocities, accelerations and joint moments calculated from the kinematic measurements were shown to be promising parameters in predicting slip severity and assessing the mechanisms (Liu and Lockhart 2006; Beschorner and Cham 2008; Hu and Qu 2013). Whole body and upper body responses to a slip incident were summarised by Liu, Lockhart, and Kim (2014) and Cham, Beschorner, and Redfern (2007).

### 4.2. Balance and stability

Nonlinear dynamics has also been used to investigate walking dynamic stability measured with accelerometers on a treadmill or a normal walking path (Stergiou 2004; Dingwell and Kang 2007; Lockhart and Liu 2008; Bruijn et al. 2013; van Schooten et al. 2013)*.* Instead of treating each step as an independent event, body movements for several consecutive steps were analysed to quantify variations in the temporal domain. The maximum Lyapunov exponent was identified as a measurement of stability (Dingwell et al. 2001; Stergiou 2004; Lockhart and Liu 2008). Further research is needed to assess the effects of working tasks and environmental conditions on dynamic stability, and to validate the relationship between dynamic stability and fall accidents.

### 4.3. Trips when walking

Stochastic distributions of the minimum foot clearance during mid swing of repeated walking of the same participant on a treadmill were investigated by Begg, Best, and Taylor (2007) and the probability of a trip event at different obstacle heights were calculated from these stochastic distributions. Walking on a treadmill could be very different from walking on an actual walkway. Therefore, it might be worthwhile to repeat the experiments by Begg, Best, and Taylor (2007) to measure the stochastic distributions of the minimum foot clearance in mid swing on an actual walkway.

For trips that occurred in early swing and late swing phases, common responses were an elevating strategy of the swing limb to overtake the obstacle and a lowering strategy to shorten the step length, respectively (Eng, Winter, and Patla 1994; Schulz 2011). The results from Grabiner et al. (1993) and Owings, Pavol, and Grabiner (2001) indicated that a recovery from a trip depended on factors such as the lower extremity muscular power, ability to restore control of the flexing trunk, reaction time, step length and walking speed. The results reported by Pijnappels, Bobbert, and van Dieën (2005a, 2005b, 2005c), showed that lower limb strength could be a critical factor in trip recovery observed in laboratory situations, thus strength training might help reduce fall risk. The heights of the obstacles used in these experiments were 5 to 15 cm. In practice, interventions, such as a ramp, are needed when the change in height of the walking surface is higher than 0.63 cm (Di Pilla 2003). Therefore, the obstacles used in these experiments could be too high to reflect what might actually be encountered in workplace settings. There is a need to systematically investigate human responses to obstacles of various heights likely to be encountered in actual workplaces.

### 4.4. Work pace (walking speed)

Typical industrial tasks require workers to perform at a greater work pace than normal walking pace. Under these circumstances, one must override the natural frequency and consciously force cadence to a faster rate to increase the walking speed. An increase in walking velocity usually increases the friction demand and risk of slip initiation (Chang, Matz, and Chang 2012). Dingwell et al. (2001) also observed that increased walking speed reduces dynamic walking stability. Neuromuscular response must be faster at greater walking velocities to accommodate the quicker time sequences of fast walking. A perturbation or error at high velocity has greater momentum than at low velocity, and requires a larger neuromuscular response to correct and stabilise the system. Therefore, faster work pace or walking speed during rushed industrial activities may adversely affect STFL initiation and balance recovery processes.

### 4.5. Load carrying

Occupational load carrying tasks are considered as one of the major factors contributing to slip and fall injuries and a causal factor leading to more than 30% (54,792 cases in 2001) of all non-fatal occupational slip and fall injuries resulting in one or more days away from work (Courtney and Webster 2001). In normal walking, corrective postural movements are made by the upper body, arms and shoulders. Arm swing is used to offset the rhythmical acceleration and deceleration of the trunk by the leg movements, and also to damp-out the rotational forces of the trunk (Haywood 1986). However, these dampening effects are modified during load carrying (Davis 1983b) and may influence risk of slip initiation (Liu, Lockhart, and Kim 2014).

### 4.6. Footwear

The human foot is the only source of direct contact with the floor during normal ambulation and plays an important role in maintaining dynamic stability (Chiou, Bhattacharya, and Succop 1996). As such, footwear may influence progression of the body during ambulation and may influence dynamic stability. For example, work shoes (e.g. stiff boots) can influence normal kinematics and kinetics at the ankle and may influence walking stability and even require more friction and increase the slip severity (Cikajlo and Matjačić 2007). Although softer footwear may allow for a better range of motion and push-off power generation, further research is needed to determine the effects of various work boots (metatarsal boots, safety-toe boots, etc.) on walking stability and comfort.

### 4.7. Ageing workforce

In order to develop effective engineering interventions and/or human support through training, the older age population segment needs to be included in the work system design. In general, isometric muscle strength peaks in the mid-twenties and then decreases slowly until after 50 years of age when there is an accelerated decline (Astrand and Rodahl 1986). Studies suggest that age-related changes in muscle strength have an important effect on recovery from a slip (Lockhart, Smith, and Woldstad 2005). This effect can be further aggravated by fatigue, and increase the risk of falls among older workers (Zhang, Lockhart, and Soangra 2015). Gait instability, sensory degradation and diminished rapid torque development capacities of the older workers imply that age must be considered as a factor in the identification of risk of occupational falls.

### 4.8. Conclusion

In conclusion, slip/trip-induced fall accidents have been investigated by various researchers utilising normal walking conditions and slip-perturbation methods. These investigators have collectively identified that the risk of slip-induced fall accidents is associated with friction demand characteristics during walking. Friction demand characteristics are affected by task factors (e.g. working-pace, turning, load carrying, etc.) as well as footwear dynamics. As such, further investigations are warranted to assess the effects of footwear properties (e.g. ankle support, personal protective equipment, shoe-sole materials, etc.) on friction demand and various industrial activities. Although initiating circumstances are important to modulate fall risks, given a perturbation (i.e. slip/trip), most investigators agree that reactive recovery characteristics are directly linked to fall severity. In other words, although slip initiating risks are directly linked to friction demand characteristics, overall fall risk is directly linked to how we maintain dynamic stability given a perturbation. Thus, concerted efforts are needed to control initiating circumstances as well as improving reactive recovery scenarios – e.g. since maintaining dynamic balance requires the upper body as well as the lower body, tasks such as carrying a load may further increase the risk of slips and falls.

## 5. Slipperiness perception

In addition to the biomechanics of STFL, researchers have considered the psychological processes involved. This has been predominately with respect to slipping. The perception of slipperiness may be psychophysical in nature (Strandberg, 1985). The role of these processes was underlined by Courtney et al. (2013), who showed that perception of slipperiness and the subsequent rate of slipping were strongly associated. Their results suggest that safety professionals could utilise aggregated worker perceptions of slipperiness to identify slipping hazards and to assess possible intervention effectiveness.

### 5.1. Proprioceptive feedback

During walking, one is often not fully aware of the fact that sliding or creep between the footwear and the floor occurs in the very beginning of the heel contact phase on contaminated surfaces and even on dry non-slippery surfaces (Perkins 1978, Strandberg and Lanshammar 1981, Perkins and Wilson 1983). The results from Leamon and Li (1990) indicated that any slip distance less than 3 cm would be detected by humans in only 50% of the occasions, and that a slip distance more than 3 cm would be perceived as a slippery condition.

When potentially hazardous conditions are perceived through visual and proprioceptive sensation, or expected to exist in the walking person’s perceptual field, walking gait is adjusted accordingly (Ekkebus and Killey 1973, Swensen, Purswell, and Schlegel 1992, Cham and Redfern 2002b, Chambers, Perera, and Cham 2013). Increases in stance and stride times and step width, as well as decreases in stride length, walking speed, heel horizontal velocity, heel horizontal and vertical accelerations, foot and floor angle and utilised coefficient of friction (UCOF) are used to avoid a slip on slippery surfaces (Swensen, Purswell, and Schlegel 1992, Bunterngchit et al. 2000, Fong, Hong, Li 2005, Lockhart, Spaulding, and Park 2007, Menant et al. 2009, Cappellini et al. 2010, Chang et al. 2015). The results from Chang et al. (2015) show that the participants in their experiment appeared to rely on the potential for foot slip, i.e. the difference between UCOF and ACOF, to form their perception of slipperiness rating under wet conditions. In addition, some kinematic variables also became major predictors of the perception of slipperiness rating under glycerol conditions. It would be beneficial to identify additional factors contributing to the perception of slipperiness and how the perception of slipperiness contributes to human responses such as kinematics and UCOF when walking on slippery surfaces.

### 5.2. Tactile sensation

In contrast to the proprioceptive feedback outlined in the previous section, tactile sensation covers the aspects of special movements performed by participants in assessing slipperiness which might not occur in daily lives. Human subjects seem to be capable of differentiating the slipperiness of floors (Yoshioka et al. 1978, 1979, Swensen, Purswell, and Schlegel 1992, Myung, Smith, and Leamon 1993, Chiou, Bhattacharya, and Succop 1996) and footwear (Strandberg 1985, Tisserand 1985, Nagata 1989, Grönqvist, Hirvonen, and Tuusa 1993) in dry, wet, or contaminated conditions. Yet Cohen and Cohen (1994b) reported significant disagreements between the measured ACOF values of tiles and subjective responses obtained by sliding bare feet across 22 test tiles under dry conditions in comparison with a standard tile with a ACOF of 0.5. According to the results from Cohen and Cohen (1994a), touch by running a bare foot across the tiles was the best sensing mechanism among touch, vision and hearing by dragging fingernails across the tiles, compared with the measured ACOF values.

Chiou, Bhattacharya, and Succop (2000) reported findings of workers’ perceived sense of slip during a standing task performance (e.g. a lateral reach task) and further related their sensory slipperiness scale to subjects’ postural sway and instability. They found that workers who were cautious in assessing surface slipperiness had less postural instability during task performance. Li, Yu, and Zhang (2011) asked participants to touch and slide across five floors with their index fingers, palms and bare feet. They reported that both index finger and palm were more sensitive than the foot in the sensation of floor roughness, and the floor surface roughness parameter was a better predictor of perceived floor slipperiness than the ACOF of the floor.

Although most people generally do not use their bare feet to sense floor slipperiness, various tactile cues can be used by safety professionals to assess slipperiness when friction measurements are not possible. The most common tactile cues used are finger touching and shoe bottom rubbing. It would be useful to compare the consistency of the results based on these tactile cues with the measured ACOF.

### 5.3. Vision

The visual field is an important psychophysiological parameter involved in gait regulation and visual impairment can lead to gait disturbance and loss of balance. Studies of the human visual mechanism have indicated that the visual field of a walking person is dynamically changing and only a small part of the effective visual field is attended to (Reed-Jones, Reed-Jones, and Hollands 2014). Therefore, if a slippery condition is not detected within one’s effective visual field (usually 3 to 4.6 m ahead), the likelihood of fall incidents is significantly increased (Zohar 1978). The involvement of visual impairment in STFL was demonstrated by Bentley et al. (2005), where underfoot hazards were not detected immediately prior to the incident in 65% of cases they studied. The causes of not being able to detect these hazards were concurrent visual task (45%), obscured view of hazard by object being carried (13%), insufficient illumination and weather condition (2%) and inability to recognise hazard (5%).

Joh et al. (2006) reported that people rely on ‘shine’ information in forming judgements of slipperiness under dry conditions despite variations as a function of surface colour, viewing distance and lighting conditions. Lesch, Chang, and Chang (2008) asked participants to rate 38 different floor surfaces under dry conditions in terms of slipperiness, reflectiveness, light/dark, traction, texture and likelihood of slipping just by looking at them. They reported that reflectiveness had the strongest correlation with perceived slipperiness (*r* = 0.73, *p* < 0.05), and slipperiness ratings correlated most strongly with the measured ACOF (*r* = −0.58, *p* < 0.05). All these studies were carried out under dry conditions, but most slip incidents occur under slippery conditions (Courtney et al. 2001b). It is important to extend these studies to more dangerous conditions under which slip incidents are more likely to occur.

Visual control of locomotion has been classified into both avoidance and accommodation strategies (Patla 1991). Avoidance strategies include, for instance, changing foot placement, increasing ground clearance, changing the direction of gait and controlling the velocity of the swing foot. Redfern and Schuman (1994) emphasised that temporal control is as critical as spatial control in placement of the foot to maintain balance during gait. Accommodation strategies involve longer term modifications, such as those outlined in section 5.1 on a slippery surface. The effects of visual cues on biomechanical strategies to manoeuvre across contaminated floor surfaces, and the effectiveness of these strategies on various surfaces, could be important in reducing STFL. Also, training as a strategy to improve the perception of slipperiness, in particular for older individuals, should be explored as suggested by Bentley and Haslam (2001a). However, we emphasise that training should not be considered without also deploying other risk elimination and reduction approaches.

### 5.4. Other intrinsic and extrinsic influences on perception

Extrinsic and intrinsic factors that can contribute to fall-related injuries are outlined by Gauchard et al. (2001). These same factors could also contribute to the perception of slipperiness. It has been demonstrated extensively how contaminants affect the perception of slipperiness. It was summarised earlier that vision could play a role. In addition, occupational organisational factors such as activities, temporal constraint and urgency, and environmental factors such as ground conditions, footwear, lighting and cold temperatures could also affect the perception rating. Intrinsic factors such as ageing, chronic or acute pathologies, alcohol, drugs, perimenopausal period, experience (including previous experience of STFL), attention, physical status, weakness and fatigue could also affect the perception rating. The results from Courtney et al. (2006) showed that a recent workplace history of slip, as well as the presence of shoe contaminants and age, could affect the perception rating. As it appears that the perception rating is a complex issue, the effects of additional factors should be explored such as demographic factors, fall history and culture.

### 5.5. Perceived slipperiness and objective measurements

The relationship between perception rating and measured coefficient of friction has been widely explored. Under laboratory or controlled environments, the perception rating has been mostly correlated with dynamic coefficient of friction (Tisserand 1969, Harris and Shaw 1988, Swensen, Purswell, and Schlegel 1992, Myung, Smith, and Leamon 1993, Grönqvist, Hirvonen, and Tuusa 1993). Likewise, the same relationship also has been reported in results from field environments, including fast food restaurants (Chang et al. 2004c, 2006, 2008), college campuses (Li et al. 2004) and a fish market (Hsu and Li 2010). Grönqvist, Hirvonen, and Tuusa (1993) reported a significant correlation between the subjective scores of slipperiness and slip distance.

## 6. Tribology

### 6.1. Friction variation

Friction has been shown to have a direct correlation with the perception of slipperiness as summarised in the previous section. Levels of ACOF are typically used to assess the potential risk of slip and fall incidents that are generally assumed more likely to occur on floors with a low ACOF. The potential for slip and fall incidents can be increased by local variations in friction due to unexpectedly encountering an abrupt reduction in friction across floor surfaces (Strandberg 1985; Pater 1985; Andres, O’Connor and Eng 1992; Grönqvist et al. 2001). Chang et al. (2008) conducted a field study in fast food restaurants and obtained various friction reduction variables that could be derived from friction measurements across each working area. They reported that two of the friction reduction variables that they evaluated could have a slightly better correlation with perception rating scores (*r* = 0.34 and 0.37) than the mean ACOF of each working area (0.33). These two variables were the absolute and relative reductions in ACOF over the whole working area where the change in ACOF was assessed in the same direction at the distance of 60 cm, approximately a step length. The role of a sudden friction change in the measurement of slipperiness, as well as the risk of slipping, should be more systematically studied with more definite results obtained in laboratory environments to provide stronger evidence for such a link.

### 6.2. Footwear tread pattern

Footwear plays a key role in slip and fall incidents as indicated in the biomechanics section. Tread patterns on shoe surfaces, in particular, affect friction, especially when surfaces are contaminated with solid particles or liquid. SATRA published guidelines for selecting proper tread patterns on shoe soles (Wilson, 1990). Li and Chen (2005) and Li, Wu, and Lin (2006) investigated the effects of tread pattern width, orientation and depth on friction measured with a portable slipmeter, the Brungraber Mark II. All of their results showed that the measured ACOF was significantly affected by the tread depth, width and orientation. The footwear pads with grooves perpendicular to the friction direction had a higher ACOF than those with parallel grooves under wet conditions (Li and Chen 2005; Li, Wu, and Lin 2006). Blanchette and Powers (2015) carried out a similar study with a whole shoe tester (SATRA STM 603) and reported that an oblique orientation with 3-mm width and 2-mm depth had the highest measured ACOF under wet conditions, while an orientation parallel to the direction of friction measurement with a 6-mm width and 6-mm depth had the lowest measured ACOF. The fundamental issues on the tread pattern selections are not well demonstrated in the published literature. The guidelines recommended by SATRA (Wilson 1990) were published without supporting scientific data. Li and Chen (2005), Li, Wu, and Lin (2006) and Blanchette and Powers (2015) evaluated simple tread patterns with single direction straight grooves, while the tread patterns of most of the footwear available in the market have more complicated geometries. Therefore, tread pattern evaluations should be expanded to include patterns with more complicated geometries such as those which are available in the market today. Singh and Beschorner (2014) reported that high fluid pressures were observed in the absence of tread and the presence of high viscosity fluids and fluid pressures were negligibly small when at least 1.5 mm of tread depth was present or when a low viscosity fluid was present. It would be desirable to conduct systematic footwear research for industries in which slip and fall injuries are significant, such as in construction and food service.

### 6.3. Wear of floor and footwear

Wear of floor and footwear is another issue that could affect friction and is important in determining the effectiveness of selections as well as potential interventions for floor and footwear to reduce slip and fall injuries. Kim (2015) investigated changes in shoe surface roughness and wear mechanisms during repeated sliding under dry conditions. They reported that progressive wear was initiated by ploughing of the floor asperities on shoe sole surfaces after only a few slidings, which was followed by simultaneous ploughing and abrasion. They also quantified wear of footwear surfaces during repeated slidings. One of the difficulties in investigating wear is that the time spans can be quite long for real-life observations. Accelerated wear methodologies that could resemble repeated actual shoe and floor interaction are needed to shorten the observation periods. In the investigations conducted by Kim (2015), shoes were rubbed against floors under dry conditions with sliding speeds that could happen only on slippery surfaces. Therefore, their results and wear mechanisms identified might not reflect what actually happens at the shoe and floor interface. Before such accelerated wear methods are established, traditional field observations to monitor shoe and floor wear, such as the studies carried out by Leclercq and Saulnier (2002) and Grönqvist (1995), should be used to develop basic understandings as a reference for the accelerated tests.

### 6.4. Surface texture of floor and footwear

Surface textures on nominally flat floor and shoe surfaces have been shown to influence friction at the shoe and floor interface under liquid contaminated conditions (Chang et al. 2001c). Surface roughness parameters representing the surface void volume, surface slope and kernel roughness depth on floor surfaces in general had strong correlations with the measured ACOF with liquid contaminants (Chang et al. 2001c, 2004b). Furthermore, Chang et al. (2004a) extended the scope to include surface waviness and identified additional surface waviness parameters that had strong correlations with the measured friction. The microscopic features on floor surfaces identified by Chang et al. (2004a, 2004b) that were related to the friction measured under liquid contaminated conditions should be studied when subjected to traffic in real-life situations. Durability of these preferred microscopic features on floor surfaces should be investigated in future research as a part of the effort to identify those features that will be able to maintain a higher friction over time.

Similar to the floor surfaces, friction increases as the roughness level on footwear is increased (Rowland, Jones, and Manning 1996; Manning and Jones 2001; Kim 2015; Mohan, Das, and Sundaresan 2015). Although Kim measured a surface parameter representing the surface void volume, most of the parameters measured revealed little about surface features such as the centre line average, maximum height, maximum depth and maximum roughness depth. Parameters that represent surface slope could be important indicators to reflect viscoelastic properties of footwear materials. A surface roughness measurement is typically carried out with a stylus profilometer. The footwear materials could deform during the measurements. It is important to investigate the effect of the stylus force on the measurements. As three-dimensional surface microscopes such as a laser scanning confocal microscope and atomic force microscope, have been used to measure footwear surfaces by Kim (2015) and Mohan, Das, and Sundaresan (2015), studies on surface parameters based on three-dimensional measurements could reveal more details about important surface features.

### 6.5. Friction measurement devices

Mechanical devices have been widely used to measure various types of friction at the shoe and floor interface (Chang et al. 2001a). A friction measurement device is intended to simulate a slip when operated on surfaces with or without contaminants in order to measure the maximum coefficient of friction that exists at the shoe and floor interface. Although human movements during slip incidents have been reported in the literature (Perkins 1978; Lanshammar and Strandberg 1983; Cham and Redfern 2002b), design and reproducibility issues for the construction of friction measurement devices necessitated some simplifications in shoe movements compared with the experimental observations. More drastic simplifications were made with portable slipmeters than with laboratory-based devices due to constraints of weight and portability, with the consequence of limited fidelity to the actual shoe and floor interface. These simplifications further resulted in significant differences in the results measured with various devices, including both laboratory-based and field-based devices (Chang et al. 2001a). Moreover, there appear to be regional preferences around the world regarding which devices could best give meaningful and useful results, so debates about their validities continue. These problems further complicate efforts in the development of interventions evaluated with friction measurements. As pointed out by Chang et al. (2001a, 2001b), the measurement conditions of these devices are still far from perfect as compared with the biomechanical data reported in the literature and are inconsistent across various devices. Bio-fidelity of these devices remains a critical issue. Does the movement of the test foot used in these devices resemble that of shoes at the critical instants of slip events? In addition to the bio-fidelity, tribo-fidelity could be more important for field-based devices. Are the tribological phenomena at the shoe and floor interface of slip events reflected at the measurement interface with these devices? Due to the requirement of portability, the contact force applied with the portable slipmeters would not be as high as that at the actual shoe and floor interface. In order to maintain the same contact pressure and lubrication conditions, the contact area and contact velocity need to be altered. Therefore, tribo-fidelity could be more important than bio-fidelity to properly reflect what actually happens at the shoe and floor interface under lubricated conditions when using these portable devices to measure friction. In the light of these limitations, further investigations are needed to identify critical instants during slip events and understand the tribological phenomena at the shoe and floor interface.

### 6.6. Solid contaminant

Scientific investigations on the operating protocols and performance of slipmeters have focused on surfaces with liquid contaminants. Solid contaminants, such as sand, sugar or flour particles, could be a slip hazard. Friction measurements on surfaces covered with sand particles was investigated by Li et al. (2007). Mills, Dwyer-Joyce, and Loo-Morrey (2009) measured friction with various particulate contaminants of different diameters (calcite and silicon) and shape factors and floors with different roughness values. They reported that the adhesive friction is significantly affected by particulate contaminants while the hysteretic component is not. They identified three lubrication mechanisms as sliding, shearing and rolling. Li, Meng, Zhang (2014) investigated the effect of different sizes of silica particles on friction under dry and wet conditions. They reported that silica particles either increased or decreased friction under dry conditions, depending on the footwear material and particle size. The silica always increased friction under wet conditions measured with Neolite and ethylene vinyl acetate (EVA). Similar investigations should be conducted on surfaces covered with other solid contaminants that are more commonly found in occupational settings.

### 6.7. Friction modelling

Friction modelling has been widely used in tribology, but is a new approach to investigating friction at the shoe and floor interface and can complement experimental approaches. Beschorner et al. (2009) developed a friction model for steady sliding between shoe and floor interface. This model was based on mixed-lubrication and included elements such as lubrication and asperity contact. Their prediction of the ACOF had good agreement with experimental measurements. Recently, a viscoelastic friction model under dry condition and steady state sliding was developed (Moghaddam, Redfern, and Beschorner 2015). However, one of the critical issues involved with friction modelling for a slip event is that the viscoelastic model should be combined with other friction mechanisms (e.g. adhesion) for the shoe sole materials. Additional issues are the non-stationary solid and liquid interface caused by the deformation of solid surfaces under boundary and hydrodynamic lubrication between liquid contaminant and shoe or floor, and transient motions involved in slip and fall incidents.

### 6.8. Slip prediction

When RCOF for an activity exceeds ACOF at the interface, a slip may happen (Redfern et al. 2001). Hanson, Redfern, and Mazumdar (1999) developed a logistic regression model to estimate slip probability in which actual fall incidents were compared with the differences between mean ACOF and mean RCOF. Both RCOF and ACOF have random variations, so the mean values used by Hanson, Redfern, and Mazumdar (1999) were inadequate for estimating the slip probability since the stochastic distributions were simply represented by their mean values. The statistical model of comparing the stochastic distributions of ACOF and RCOF introduced by Chang (2004) is a promising way to estimate the probability of slip incidents when unexpectedly encountering a low friction area. Chang et al. (2012) reported that the distribution of the RCOF appears to have a good match with the normal distribution for most of the conditions in their experiment (85.5%), but each foot had a different distribution from the other foot under the same conditions in 76% of cases. Chang, Matz, and Chang (2014) investigated the stochastic distributions of the ACOF of five floor surfaces under dry, water and glycerol conditions. They reported that the ACOF distributions had a slightly better match with the normal and log-normal distributions than with the Weibull in only three out of 15 cases evaluated. Since the ACOF was compared with the RCOF for the estimate of slip probability, the distribution of the ACOF in seven conditions out of 15 could be considered a constant for this purpose when the ACOF was much lower or higher than the RCOF. No representation could be found in three conditions out of 15. Further investigations could be conducted in the future on the effects of ageing, anthropometric distribution on the stochastic distribution of RCOF, as well as the stochastic distributions of ACOF of commonly used floor surfaces. Ultimately, the output of the statistical model should be validated by experiments and the results considered valid only when ACOF measured with adequate fidelity is compared with RCOF.

### 6.9. Floor cleaning

Floor cleaning has received very little attention despite the efforts by Underwood (1991) and Quirion, Poirier, and Lehane (2008). Underwood (1991) analysed soil on typical floor tiles on restaurant floors and then proposed a process to generate a worn and soiled tile in the laboratory. The worn tiles generated by the process used by Underwood (1991) were not compared with actual worn tiles in terms of surface roughness and material compositions. The chemical compositions and structures of the contaminants on the fouled tiles generated by their process were not compared with contaminants on actual worn tiles. On top of fouled tiles, fresh contaminants such as cooking oil were applied by Quirion, Poirier, and Lehane (2008). These fresh contaminants might not resemble those contaminants deposited on fouled tiles in field environments.

Quirion, Poirier, and Lehane (2008) used the techniques developed by Underwood and onsite cleaning procedures observed in field visits to investigate the effectiveness of floor cleaning and improve cleaning protocols for various floor types. They reported that the cleaning efficiency and friction could be improved by simple changes in floor cleaning procedures. In addition, proper executions of existing cleaning protocols could affect the outcome. Verma et al. (2010) reported that 62% of the participants who were responsible for cleaning floors used hot/warm water with widely used enzyme-based floor cleaners, thus violating the manufacturer’s cold water floor cleaning protocol. Quirion, Poirier, and Lehane (2008) used very limited cleaning methods with few cleaning chemicals in their experiment. Systematic studies are needed to identify optimal cleaning methods and chemicals used to achieve the best cleaning outcomes in a real-world setting.

## 7. Organisational influences

It should be noted that caution is needed when translating findings of laboratory studies to activities in actual workplaces. Walking and movements performed at work and the injury risks arising from them are determined by working conditions (e.g. work-pace, load carriage), worker characteristics (e.g. obesity, age) and worker goals (e.g. stress, motivation). A comprehensive understanding of balance and movement control in occupational situations requires consideration of not only the biomechanics of movement, but also the cognitive, psychological and organisational aspects (Leclercq et al. 2015). Indeed, displacements and, more generally, the movements performed at work are subjected to continuous adjustment of the required task as well as individual, organisational and environmental constraints. Thus, time required and time imposed for the action (i.e. workload as well as work-pace), mistakes made (Chassaing 2005, 2010), tiredness, pain (Gaudart 2000; Derosier et al. 2008), previous work experience, life outside work (Chassaing 2005; Derosier et al. 2008; Caban-Martinez et al. 2014) and past experiences (Daniellou et al. 2008) are a few factors influencing our movement/motor patterns relevant to occupational fall risks. Moreover, production-safety arbitrations, which relate particularly to organisational activities implemented by the company, should be considered. Organisational factors highlighted during STFL analysis reveal worker arbitration between production and safety in the work situation, in which he/she is exposed to a risk of displacement or movement disturbance (Leclercq 2014).

### 7.1. Developing a systems approach

Various authors have emphasised the role of organisational influences on worker exposure to STFL hazards and eliminating or controlling risks (Bentley and Haslam 2001b; Leclercq and Thouy 2004; Derosier et al. 2008; Leclercq 2014; Leclercq et al. 2015), while others argued explicitly for a systems or macro-ergonomics approach to STFL prevention (Leclercq 2002; Gao and Abeysekera 2004; Maynard and Robertson 2007; Bentley 2009). The reasoning for a systems approach is that for any STFL incident, its genesis will lie within the context of a socio-technical system, i.e. the combination and interactions among humans, equipment, work activities, environments, organisational structures and processes all affecting workplace safety (Carayon et al. 2015). It follows that it is necessary to develop scientific rationales considering worker attributes, work tasks, interactions with the physical environments and organisational factors to explain the processes involved in STFL.

Immediate or proximal factors in STFL, such as slippery flooring, inadequate footwear, the presence of trip hazards and unsafe behaviour are themselves caused by other upstream or distal factors. These factors could include, for example, nature of the tasks undertaken, equipment selection and usage (Bentley and Haslam 2001a; Kines 2003), work organisation (Leclercq and Thouy 2004), work system design (Derosier et al. 2008) and safety management (Bentley and Haslam 2001b). For example, Bentley and Haslam (1998) showed that the ‘job and finish’ policy implemented in the United Kingdom’s mail distribution company, which at that time allowed workers to go home as soon as the last mail had been distributed, could have encouraged workers to take risks by hurrying or taking short-cuts. Each factor involved in an STFL incident, regardless of its position in the causal chain, represents an opportunity, theoretically at least, for its prevention.

In the case of distal factors, their presence and combination will be specific to an organisation, industrial sector or occupation. This is illustrated by STFL studies in the literature concerned with particular work activities or work situations including delivery driving (Nicholson and David 1985), painting (Hunting et al. 1991), postal delivery (Haslam and Bentley 1999), power distribution (Leclercq and Thouy 2004; Mattila et al. 2006), health care (Staal et al. 2004; Bell et al. 2008), dairy farming (Bentley et al. 2005), seafaring (Jensen et al. 2005), rail transport (Leclercq, Thouy, Rossignol 2007), metallurgy (Derosier et al. 2008), metallurgy and construction (Abdat et al., 2014) and helicopter manufacturing (Amandus et al. 2012). Such investigations reveal a particular socio-technical system context and pattern of causal factors related to this socio-technical system. Slip-induced falls are a particular problem in restaurant workplaces, for example. Flach et al. (2015) considered slips in a national chain of fast food restaurants from a socio-technical systems perspective, focusing on the influences on a single factor (floor cleanliness). Flach et al.’s analysis showed how floor cleaning and its effectiveness were affected by organisational practices and decision-making, such as choice of detergent by the head office, improper use of the chosen detergent locally and the role of line communication and supervision. Flach et al. also identified the challenge for the company in maintaining local knowledge and correct floor cleaning practices in an industry with high turnover of staff and low paid workers.

In their commentary on STFL prevention, Maynard and Robertson (2007) referred to macro-ergonomics as an implementation of socio-technical system approaches and then proceeded to describe a work-system continuum. Key elements in this work-system continuum included management leadership, education and training, hazard surveillance, floor slipperiness assessment, incident and injury reports, floor surface selection, floor surface treatments, mats, housekeeping and maintenance, warning signs and instructions, and slip-resistant footwear. Maynard and Robertson concluded that preventing STFL requires a multi-factorial approach and combined effort among all members of an organisation, with communication across the entire work system being critical.

### 7.2. Safety climate and STFL

When considering organisational influences on STFL, another relevant concept is safety climate. Safety climate is defined as workers’ shared perceptions of safety policies, procedures and practices, as well as the overall importance and priority given to safety at work by their organisation and in their workplace (Zohar 2003). It has also been suggested that safety climate could be related to workers’ perceptions of injury risk (Mearns and Flin 1996). Safety climate, a multi-factorial construct, has been shown to be a robust predictor of safety outcomes, such as incidents and injuries, across industries and countries (Huang et al. 2007; Zohar 2010). There has, however, been only limited attention given to the relationship between safety climate and STFL.

Bentley and Haslam (2001a) examined safety climate indirectly in their comparison of safety practices of managers of high and low accident rate postal delivery offices. They found that delivery office managers from low accident rate offices, drawn equally from matched high and low accident rate offices, appeared to have improved performance in quality of safety communication, dealing with hazards reported on delivery walks, and accident investigation and remedial action.

Swedler et al. (2015) reported a prospective study examining the relationship between safety climate and workplace slips involving 349 workers at 30 fast food restaurants in the US. At baseline, participants were questioned about safety training and management commitment to safety at their restaurant, with responses used to generate safety climate scores. Workers’ shoes were also assessed for slip resistance, with this rating included as a safety performance metric. The study found that safety climate influenced prospective slipping in restaurants, mediated by employees wearing slip-resistant shoes. Swedler et al. concluded that it should be possible to improve safety climate by improving training and managerial commitment to safety and in so doing reduce the prevalence of workplace slips. Further research is needed to confirm the role of other safety climate factors in STFL such as communication, management commitment and competing demands. Additional research issues could include the potential of safety climate measures being more widely used as a tool for evaluating STFL risk in organisations, and the possibilities for improving safety climate as a means of STFL prevention.

## 8. Injury prevention and practices

As is apparent from earlier sections of this paper, the causes of STFL have been the focus of substantial research effort. Research with regard to STFL prevention, however, is another matter. There has been attention in the falls literature to specific hazards and to controlling the associated risks. From various research studies, for example, it is known that proper selection of footwear and flooring, considering the nature of any floor surface contamination that may occur, can increase the friction at the foot-floor interface thus reducing slips (e.g. Aschan et al. 2009; Verma et al. 2011). Based on their prospective cohort study conducted in fast food restaurants, Verma et al. (2011) showed that the use of slip-resistant shoes was associated with a 54% reduction in the reported rate of slipping. They also showed that the rate of slipping decreased as the mean kitchen coefficient of friction increased. A note of caution with footwear-based interventions is that, in occupations with variable underfoot conditions and task requirements, specifying appropriate footwear for the range of conditions that may be encountered presents challenges (e.g. Manning and Jones 2001; Aschan et al. 2005).

There remain notable gaps in our knowledge on STFL prevention. For example, knowledge of how floor cleaning protocols can reduce floor slipperiness is underdeveloped. The level and character of lighting that is needed in order to move around and negotiate the environment safely from a falls perspective is only crudely understood. There have been limited studies of the effectiveness of training, education and awareness raising as an approach to STFL reduction. These studies covered a slip-simulator to facilitate kinetic learning (Lockhart 2010; Rich 2012), and a virtual reality platform (Liu et al. 2015; Parijat, Lockhart, and Liu 2015a, 2015b).

It is significant from an ergonomics perspective that there has been only limited research adopting an ergonomics systems approach, addressing the ‘… important latent failures or the upstream organisational and cultural contexts within which workplace STF occur’ (Bentley, 2009). Noting the lack of progress with STFL prevention, Leclercq et al. (2015) noticed the similarity between work-related musculoskeletal disorders (WMSD) and slips, trips and falls, resulting from movements made by workers. Leclercq et al. (2015) argued that STFL prevention could benefit from the extensive research effort directed at WMSD, at least from a methodological and theoretical point of view. From this, they identified the benefits in developing STFL prevention approaches based on a local, participatory search for solutions that take into account mental representations of the risk shared by all stakeholders.

Another surprising aspect is the paucity of prospective studies and evaluated occupational STFL prevention intervention programmes. This lack can be contrasted with the major effort addressing falls among older people. Gillespie (2013), a longstanding author of the Cochrane reviews on interventions for prevention of falls among older people, reported that as of 2012, there had been over 200 randomised controlled trials, involving almost 140,000 participants, addressing falls prevention among this group. An aspect with this is that prevention of falls among older people has often been focused on the mitigation of causal factors linked to individuals having greater susceptibility to falling. With causal patterns being different and more diverse for occupational STFL, prevention strategies are probably more difficult to define and to implement.

In the area of STFL, however, the only evaluated, multi-factorial intervention is the important study by Bell et al. (2008). Their research involved three hospitals in the US, applying a comprehensive package of intervention measures, phased in over 3 years and then monitored during a 3-year post-evaluation period. The intervention measures, which were based on analysis of the hospitals’ historical accident reporting data and on-site risk assessment, were developed around 11 main components (Table 1).

**Table 1.** Main components of Bell et al. (2008) hospital falls reduction intervention.

• Keep floors clean and dry • Prevent entry into areas that are contaminated • Use slip-resistant shoes • Keep walkways clear of objects and reduce clutter • Provide adequate lighting in all work areas including outdoor stairwells and parking garages • Secure loose cords, wires and tubing • Eliminate outdoor surface irregularities • Eliminate indoor surface irregularities • Check stairs • Prepare for ice and snow • General awareness campaign

The results from the Bell et al. (2008) intervention measures showed that the overall workers compensation STFL injury claims rate for the hospitals declined significantly (over 50%) during the post-intervention time period. A major success of the intervention showed that a comprehensive and sustained intervention can have a major effect in reducing STFL and related injuries. What the study was unable to reveal, however, was the relative effect or interdependency of the intervention components.

Drawing on the current knowledge that is available in the literature, a structured risk management approach to STFL reduction and injury prevention is appropriate. This approach was the starting point for the tailored strategy of Bell et al. (2008) for their hospital intervention. Similarly, Haslam and Stubbs (2006) described a generic approach with three overarching components: (i) primary prevention, (ii) residual risk reduction and (iii) measures to maximise individual capability, as outlined in Table 2 and expanded upon in the following sections.

**Table 2.** Prevention measures for STFL (adapted from Haslam and Stubbs, 2006).

Primary Prevention	Risk Reduction	Maximised Capability
• Provide slip resistant flooring • Design work/activity systems to avoid presence of fall risks (attention to environments, equipment, layouts, tasks and people) • Cover outside walkways to keep off rain, snow, ice, leaves • Design walkways to exclude trip hazards • Plan pedestrian routes to allow sufficient space between individuals • Separate pedestrians from moving machinery and vehicles • Provide sufficient, convenient space for storage • Avoid need for walking/standing on surfaces that move unpredictably • Install adequate lighting • Design and select environment features to facilitate cleaning and maintenance • Design and select environment features for durability and resistance to damage	• Provide education and awareness raising of fall risks and fall consequences • Perform fall risk assessments and implement controls • Organise sustainable housekeeping procedures for inspection, cleaning and maintenance • Manage fall risks introduced during installation, cleaning and maintenance • Provide warning signs for slip hazards • Mark trip hazards • Encourage use of lighting • Discourage carrying of awkward, heavy loads • Avoid creating circumstances that encourage rushing • Implement risk management protocols for inclement weather • Implement risk management protocols for individuals at increased risk of falling	• Encourage use of suitable footwear • Encourage use of suitable clothing, including personal protective equipment (PPE) • Encourage eye tests and appropriate use of spectacles • Encourage exercise for strength, coordination and balance • Adopt occupational health protocols to minimise fall risk from prescribed medication • Consider fall risk arising from shiftworking

### 8.1. Primary prevention

The purpose of primary prevention is to eliminate STFL hazards at source through the design of the work environment and work/activity systems. Flooring should be selected with appropriate slip resistance for the different conditions to which it will be subjected. Walkways and walking areas should be designed and constructed to avoid trip hazards. In addition, primary prevention involves attention to the equipment used (e.g. to avoid spillages and other walkway contamination), the manner in which equipment is arranged, the tasks workers need to perform, and the extent to which each of these elements might affect the risk of falling. Provision of sufficient, accessible storage is a measure aimed at reducing the need for objects and materials to be placed in the walking path, which may then present a trip hazard. The provision of sufficient lighting is important to aid visibility of the walking surface. The design and installation of walking surfaces and pathways should make allowances for their cleaning and maintenance. In addition, to avoid introducing hazards by wear and tear, installations should be appropriately durable and resistant to damage. Pedestrian walkways can be protected from vehicle intrusion or damage, for example, by ensuring there is physical separation between the two (e.g. through installation of bollards).

### 8.2. Risk reduction

Even with concerted attention to primary fall prevention, it is inevitable that STFL hazards will continue to be present in the environment. Risk reduction aims to reduce the likelihood of STFL and injuries arising from these hazards. An important starting point is to raise awareness of the problem and, through education, promote understanding of risk factors for falling and how they can be mitigated. Accompanying this is a need for proactive risk assessment and management.

Where STFL hazards may arise in an area used by pedestrians, it is important that adequate procedures are implemented to detect these hazards and to remedy the situation. Indoor flooring will usually need to be cleaned periodically for the sake of hygiene and appearances. Care should be taken during the cleaning process to make sure STFL hazards are not introduced, for example, the risk of slipping with wet vinyl or tiled floor surfaces while these surfaces are drying. For maintenance, routine inspection programmes should be arranged for walking areas and pathways. In all cases, housekeeping procedures should be designed to be sustainable so that initial good practices do not deteriorate to the point of becoming ineffective, as can readily occur over time.

Where STFL hazards are present and cannot be removed immediately, an obvious action is to warn of their existence. This can be done through use of signage warning of a risk of slipping. Lighting may be adequate, but is only effective if turned on at appropriate times. Carrying items and hurrying are additional behavioural factors contributing to STFL and should be discouraged in circumstances where other STFL risk factors are present. These behavioural factors often reveal more upstream organisational and cultural factors (Leclercq 2014).

There are certain conditions in which risk of STFL is increased. Poor weather, resulting in outdoor areas becoming covered with ice or snow, is frequently accompanied by increased prevalence of slip-induced falls, unless appropriate precautions have been taken. It should be possible to plan ahead for such occasions and facility managers ought to be ready and prepared to implement measures to reduce risk, either through clearing affected areas or by reducing exposure to the slippery conditions (e.g. by temporary changes to working practices which keep workers indoors).

### 8.3. Maximised capability

A third strand of the STFL prevention process is to maximise individual ability to negotiate the workplace environment. Use of footwear commensurate with underfoot conditions is a measure that can reduce slipping. This measure should include an employer advising on and, where appropriate, issuing suitable footwear for slippery outdoor conditions and shoes or boots with special soling for indoor occupational situations where floor contamination cannot be avoided. Protective clothing can restrict movement and cause sensory impairment, as may be the case with respirators and hearing protection, for example. Protective eyewear can distort vision. Thus, consideration is needed to STFL safety when specifying and managing the use of workplace apparel.

Risk of STFL is reduced if people can see what they are doing; thus, there may be benefit in promoting regular eyesight testing among workers, along with encouragement to use spectacles appropriately. Encouraging exercise to increase and maintain strength and coordination can help improve balance as well as having other benefits in promoting workability. Certain medications that may be prescribed for individual workers for a range of common health conditions can cause drowsiness, dizziness, unsteadiness and blurred vision, all undesirable from an STFL prevention perspective. Tiredness, as may arise among shift workers, can affect concentration and attention. The effects of alcohol on coordination and balance are well known, although this is not often a problem among a well-managed workforce. There is a particular need, however, to control STFL risk in workplace locations where alcohol is consumed regularly and drinks may be spilt or drink containers discarded onto the floor (e.g. by customers in bars and clubs).

While the risk management approach advocated by Haslam and Stubbs (2006) appears intuitive and based on sound reasoning, the ‘state of science’ for STFL prevention is such that evidence in support of its various elements and their prioritisation is sparse.

## 9. Conclusions

This paper has reviewed the state of science concerning occupational STFL. The review has highlighted the continuing burden of STFL as a major source of workplace injury and subsequent cost to individuals, employers and wider society. Progress has been made in understanding the causal factors contributing to STFL, with slipping and the foot-floor interface, in particular, having received detailed attention. The contributing factors in trip-induced falls have also been examined, both experimentally and in workplace studies. Less attention has been given to same level falls arising from other loss of balance or movement disturbance events.

Although there is increasing recognition of the complexity of the interacting factors in STFL and the need for multi-disciplinary approaches, systems perspectives adopting a more holistic view of STFL causation are immature. Further work is needed, drawing on current developments in socio-technical systems thinking and safety. Greater attention is necessary to the factors upstream in the injury genesis forming the circumstances in which injuries occur. It is important to consider these upstream factors in STFL prevention.

The scale of STFL and the limited success in tackling the problem present a compelling case for further prevention trials to be undertaken in the field. Structured and evaluated studies will be essential in developing evidence-based approaches aimed at reducing the toll of STFL. Although important research has shown that intervention can be effective, resulting in reductions in the occurrence of injuries, confirmation is required regarding the effectiveness of different intervention components, both separately and combined. Given the multi-factorial nature of STFL, this improved understanding will allow intervention efforts to be better targeted and more feasible, taking into account cost effectiveness. Intervention research of this nature, with the cooperation needed by organisations and access needed to their workplaces and their workers, does present significant practical challenges. With coordinated, international research efforts, however, further progress should be possible.

In conclusion, the major messages from this state of science review are that STFL continue to be a major source of occupational injury. Progress has been made understanding the causes of STFL, with increasing recognition of the multi-factorial nature of the problem. Gaps in understanding still exist and have been flagged for further research. There is limited but encouraging evidence that STFL prevention activity can be beneficial in reducing injuries. Further research is needed to improve knowledge of the measures most beneficial for STFL prevention, how to deploy these and the cost-benefits of doing so. Finally, we underline that STFL occur in a socio-technical systems context. A systems approach will be essential to bring about real future progress in their prevention.

## Disclosure statement

No potential conflict of interest was reported by the authors.

## References

[CIT0001] Abdat F., Leclercq S., Cuny X., Tissot C. (2014). Extracting Recurrent Scenarios from Narrative Texts Using a Bayesian Network: Application to Serious Occupational Accidents with Movement Disturbance. *Accident Analysis and Prevention*.

[CIT0002] Alexander N. B., Shepard N., Gu M. J., Schultz A. (1992). Postural Control in Young and Elderly Adults When Stance is Perturbed: Kinematics. *Journal of Gerontology*.

[CIT0003] Amandus H., Bell J., Tiesman H., Biddle E. (2012). The Epidemiology of Slips, Trips, and Falls in a Helicopter Manufacturing Plant. *Human Factors: The Journal of the Human Factors and Ergonomics Society*.

[CIT0004] Anderson D. E., Franck C. T., Madigan M. L. (2014). Age Differences in the Required Coefficient of Friction during Level Walking Do Not Exist When Experimentally-controlling Speed and Step Length. *Journal of Applied Biomechanics*.

[CIT0005] Andres R. O., O’Connor D., Eng T., Kumar S. (1992). A Practical Synthesis of Biomechanical Results to Prevent Slips and Falls in the Workplace.

[CIT0006] Aschan C., Hirvonen M., Rajamäki E., Mannelin T. (2005). Slip Resistance of Oil Resistant and Non-oil Resistant Footwear Outsoles in Winter Conditions. *Safety Science*.

[CIT0007] Aschan C., Hirvonen M., Rajamäki E., Mannelin T., Ruotsalainen J., Ruuhela R. (2009). Performance of Slippery and Slip-resistant Footwear in Different Wintry Weather Conditions Measured in Situ. *Safety Science*.

[CIT0008] Astrand P. O., Rodahl K. (1986). *Textbook of Work Physiology*.

[CIT0009] Begg R., Best R., Dell’Oro L., Taylor S. (2007). Minimum Foot Clearance during Walking: Strategies for the Minimisation of Trip-related Falls. *Gait and Posture*.

[CIT0010] Bell J. L., Collins J. W., Wolf L., Grönqvist R., Chiou S., Chang W. R., Sorock G. S., Courtney T. K., Lombardi D. A., Evanoff B. (2008). Evaluation of a Comprehensive Slip, Trip and Fall Prevention Programme for Hospital Employees. *Ergonomics*.

[CIT0011] Bentley T. A., Haslam R. A. (1998). Slip, Trip and Fall Accidents Occurring during the Delivery of Mail. *Ergonomics*.

[CIT0012] Bentley T. A., Haslam R. A. (2001a). Identification of Risk Factors and Countermeasures for Slip, Trip and Fall Accidents during the Delivery of Mail. *Applied Ergonomics*.

[CIT0013] Bentley T. A., Haslam R. A. (2001b). A Comparison of Safety Practices Used by Managers of High and Low Accident Rate Postal Delivery Offices. *Safety Science*.

[CIT0014] Bentley T. A., Tappin D., Moore D., Legg S., Ashby L., Parker R. (2005). Investigating Slips, Trips and Falls in the New Zealand Dairy Farming Sector. *Ergonomics*.

[CIT0015] Bentley T. (2009). The Role of Latent and Active Failures in Workplace Slips, Trips and Falls: An Information Processing Approach. *Applied Ergonomics*.

[CIT0016] Beringer D. N., Nussbaum M. A., Madigan M. L. (2014). Temporal Changes in the Required Shoe-floor Friction When Walking following an Induced Slip. *PLoS ONE*.

[CIT0017] Beschorner K., Cham R. (2008). Impact of Joint Torques on Heel Acceleration at Heel Contact, a Contributor to Slips and Falls. *Ergonomics*.

[CIT0018] Beschorner K., Lovell M., Higgs C. F., Redfern M. S. (2009). Modeling Mixed-Lubrication of a Shoe-floor Interface Applied to a Pin-on-Disk Apparatus. *Tribology Transactions*.

[CIT0019] Blanchette M. G., Powers C. M. (2015). The Influence of Footwear Tread Groove Parameters on Available Friction. *Applied Ergonomics*.

[CIT0020] Bruijn S. M., Meijer O. G., Beek P. J., van Dieën J. H. (2013). Assessing the Stability of Human Locomotion: A Review of Current Measures. *Journal of the Royal Society Interface*.

[CIT0021] Buck P. C., Coleman V. P. (1985). Slipping, Tripping and Falling Accidents at Work: A National Picture. *Ergonomics*.

[CIT0022] Bureau of Labour Statistics (2014). Economic News Release: Table 5. Number, Incidence Rate, and Median Days Away from Work for Nonfatal Occupational Injuries and Illnesses Involving Days Away from Work by Injury or Illness Characteristics and Ownership, 2013. http://www.bls.gov/news.release/osh2.t05.htm.

[CIT0023] Bunterngchit Y., Lockhart T., Woldstad J. C., Smith J. L. (2000). Age Related Effects of Transitional Floor Surfaces and Obstruction of View on Gait Characteristics Related to Slips and Falls. *International Journal of Industrial Ergonomics*.

[CIT0024] Burnfield J. M., Tsai Y. J., Powers C. M. (2005). Comparison of Utilized Coefficient of Friction during Different Walking Tasks in Persons with and without a Disability. *Gait and Posture*.

[CIT0025] Caban-Martinez A. J., Courtney T. K., Chang W. R., Lombardi D. A., Huang Y. H., Brennan M. J., Perry M. J., Katz J. N., Verma S. K. (2014). Preventing Slips and Falls through Leisure-time Physical Activity: Findings from a Study of Limited-Service Restaurants. *PLoS ONE*.

[CIT0026] Cameron I. D., Gillespie L. D., Robertson M. C., Murray G. R., Hill K. D., Cumming R. G., Kerse N. (2012). Interventions for Preventing Falls in Older People in Care Facilities and Hospitals. *Cochrane Database of Systematic Reviews*.

[CIT0027] Cappellini G., Ivanenko Y. P., Dominici N., Poppele R. E., Lacquaniti F. (2010). Motor Patterns during Walking on a Slippery Walkway. *Journal of Neurophysiology*.

[CIT0028] Carayon P., Hancock P., Leveson N., Noy I., Sznelwar L., van Hootegem G. (2015). Advancing a Sociotechnical Systems Approach to Workplace Safety – Developing the Conceptual Framework. *Ergonomics*.

[CIT0029] Cham R., Redfern M. S. (2002a). Changes in Gait When Anticipating Slippery Floors. *Gait and Posture*.

[CIT0030] Cham R., Redfern M. S. (2002b). Heel Contact Dynamics during Slip Events on Level and Inclined Surfaces. *Safety Science*.

[CIT0031] Cham R., Beschorner K., Redfern M. S. (2007). Whole Body Postural Responses to Slips.

[CIT0032] Chambers A. J., Perera S., Cham R. (2013). Changes in Walking Characteristics of Young and Older Adults When Anticipating Slippery Floors. *IIE Transactions on Occupational Ergonomics and Human Factors*.

[CIT0033] Chang W. R. (2008). Preface. *Ergonomics*.

[CIT0034] Chang W. R., Grönqvist R., Leclercq S., Brungraber R., Mattke U., Strandberg L., Thorpe S., Myung R., Makkonen L., Courtney T. K. (2001a). The Role of Friction in the Measurement of Slipperiness, Part 2: Survey of Friction Measurement Devices. *Ergonomics*.

[CIT0035] Chang W. R., Grönqvist R., Leclercq S., Myung R., Makkonen L., Strandberg L., Brungraber R., Mattke U., Thorpe S. (2001b). The Role of Friction in the Measurement of Slipperiness, Part 1: Friction Mechanisms and Definition of Test Conditions. *Ergonomics*.

[CIT0036] Chang W. R., Kim I. J., Manning D., Bunterngchit Y. (2001c). The Role of Surface Roughness in the Measurement of Slipperiness. *Ergonomics*.

[CIT0037] Chang W. R. (2004). A Statistical Model to Estimate the Probability of Slip and Fall Incidents. *Safety Science*.

[CIT0038] Chang W. R., Grönqvist R., Hirvonen M., Matz S. (2004a). The Effect of Surface Waviness on Friction between Neolite and Quarry Tiles. *Ergonomics*.

[CIT0039] Chang W. R., Hirvonen M., Grönqvist R. (2004). The Effects of Cut-off Length on Surface Roughness Parameters and Their Correlation with Transition Friction. *Safety Science*.

[CIT0040] Chang W. R., Li K. W., Huang Y. H., Filiaggi A., Courtney T. K. (2004). Assessing Floor Slipperiness in Fast-food Restaurants in Taiwan Using Objective and Subjective Measures. *Applied Ergonomics*.

[CIT0041] Chang W. R., Li K. W., Huang Y. H., Filiaggi A., Courtney T. K. (2006). Objective and Subjective Measurements of Slipperiness in Fast-Food Restaurants in the USA and Their Comparison with the Previous Results Obtained in Taiwan. *Safety Science*.

[CIT0042] Chang W. R., Huang Y. H., Li K. W., Filiaggi A., Courtney T. K. (2008). Assessing Slipperiness in Fast-Food Restaurants in the USA Using Friction Variation, Friction Level and Perception Rating. *Applied Ergonomics*.

[CIT0043] Chang W. R., Chang C. C., Matz S. (2011). The Effect of Transverse Shear Force on the Required Coefficient of Friction for Level Walking. *Human Factors: The Journal of the Human Factors and Ergonomics Society*.

[CIT0044] Chang W. R., Matz S., Chang C. C. (2012). Stochastic Distribution of the Required Coefficient of Friction for Level Walking – An In-depth Study. *Ergonomics*.

[CIT0045] Chang W. R., Matz S., Chang C. C. (2014). The Stochastic Distribution of Available Coefficient of Friction for Human Locomotion of Five Different Floor Surfaces. *Applied Ergonomics*.

[CIT0046] Chang W. R., Lesch M. F., Chang C. C., Matz S. (2015). Contribution of Gait Parameters and Available Coefficient of Friction to Perceptions of Slipperiness. *Gait & Posture*.

[CIT0047] Chassaing K. (2005). Stratégies D’expérience Et Organisation Du Travail Dans La Prévention Des Douleurs Articulaires.

[CIT0048] Chassaing K. (2010). Les “Gestuelles” à L’épreuve De L’organisation Du Travail: Du Contexte De L’industrie Automobile à Celui Du Génie Civil. *Le Travail Humain*.

[CIT0049] Chau N., Gauchard G. C., Siegfried C., Banamghar L., Dangelzer J. L., Francais M., Sourdot A., Perrin P. P., Mur J. M. (2004). Relationships of Job, Age, and Life Conditions with the Causes and Severity of Occupational Injuries in Construction Workers. *International Archives of Occupational and Environmental Health*.

[CIT0050] Chiou S. Y., Bhattacharya A., Succop P. A. (1996). Effect of Workers’ Shoe Wear on Objective and Subjective Assessment of Slipperiness. *American Industrial Hygiene Association Journal*.

[CIT0051] Chiou S. Y., Bhattacharya A., Succop P. A. (2000). Evaluation of Workers’ Perceived Sense of Slip and Effect of Prior Knowledge of Slipperiness during Task Performance on Slippery Surfaces. *AIHAJ – American Industrial Hygiene Association*.

[CIT0052] Cikajlo I., Matjačić Z. (2007). The Influence of Boot Stiffness on Gait Kinematics and Kinetics during Stance Phase. *Ergonomics*.

[CIT0053] CNAMTS (1988). *Statistiques Nationales Des Accidents Du Travail, Des Accidents De Trajet Et Des Maladies Professionnelles*.

[CIT0054] CNAMTS (2012). *Statistiques Nationales Des Accidents Du Travail, Des Accidents De Trajet Et Des Maladies Professionnelles*.

[CIT0055] Cohen H. H., Cohen D. M. (1994a). Psychophysical Assessment of the Perceived Slipperiness of Floor Tile Surfaces in a Laboratory Setting. *Journal of Safety Research*.

[CIT0056] Cohen H. H., Cohen D. M. (1994b). Perceptions of Walking Surface Slipperiness under Realistic Conditions, Utilizing a Slipperiness Rating Scale. *Journal of Safety Research*.

[CIT0057] Courtney T. K., Chang W. R., Grönqvist R., Redfern M. S. (2001a). The Measurement of Slipperiness – An International Symposium. *Ergonomics*.

[CIT0058] Courtney T. K., Huang Y. H., Verma S. K., Chang W. R., Li K. W., Filiaggi A. J. (2006). Factors Influencing Restaurant Worker Perception of Floor Slipperiness. *Journal of Occupational and Environmental Hygiene*.

[CIT0059] Courtney T. K., Sorock G. S., Manning D. P., Collins J. W., Holbein-Jenny M. A. (2001b). Occupational Slip, Trip, and Fall-related Injuries – Can the Contribution of Slipperiness Be Isolated?. *Ergonomics*.

[CIT0060] Courtney T. K., Verma S. K., Chang W. R., Huang Y. H., Lombardi D. A., Brennan M. J., Perry M. J. (2013). Perception of Slipperiness and Prospective Risk of Slipping at Work. *Occupational and Environmental Medicine*.

[CIT0061] Courtney T. K., Webster B. S. (2001). Antecedent Factors and Disabling Occupational Morbidity Insights from the New BLS Data. *AIHAJ – American Industrial Hygiene Association*.

[CIT0062] Daniellou F., Caroly S., Coutarel F., Escriva E., Roquelaure Y., Schweitzer J. M. (2008). *“La Prévention Durable Des TMS Quels Freins? Quels Leviers D’action?” Recherche-action 2004–2007*.

[CIT0063] Davis P. R. (1983a). Slipping, Tripping and Falling Accidents: An Introduction. *Ergonomics*.

[CIT0064] Davis P. R. (1983b). Human Factors Contributing to Slips, Trips and Falls. *Ergonomics*.

[CIT0065] Derosier C., Leclercq S., Rabardel P., Langa P. (2008). Studying Work Practices: A Key Factor in Understanding Accidents on the Level Triggered by a Balance Disturbance. *Ergonomics*.

[CIT0066] Di Pilla S. (2003). *Slip and Fall Prevention*.

[CIT0067] Dingwell J. B., Cusumano J. P., Cavanagh P. R., Sternad D. (2001). Local Dynamic Stability versus Kinematic Variability of Continuous Overground and Treadmill Walking. *Journal of Biomechanical Engineering*.

[CIT0068] Dingwell J. B., Kang H. G. (2007). Differences between Local and Orbital Dynamic Stability during Human Walking. *Journal of Biomechanical Engineering-Transactions of the ASME*.

[CIT0069] Ehsan R., Bochen J., Nussbaum M., Lockhart T. (2013). Investigating the Effects of Slipping on Lumbar Muscle Activity, Dinematics, and Dinetics.

[CIT0070] Ekkebus C. E., Killey W. (1973). Measurement of Safe Walkway Surfaces. *Soap/Cosmetics/Chemical Specialties*.

[CIT0071] Eng J. J., Winter D. A., Patla A. E. (1994). Strategies for Recovery from a Trip in Early and Late Swing during Human Walking. *Experimental Brain Research*.

[CIT0072] European Agency for Safety and Health at Work (EU-OSHA) (2001). *Preventing Accidents at Work*.

[CIT0073] European Commission (2008). *Causes and Circumstances of Accidents at Work in the EU*.

[CIT0074] European Commission (2013). *European Statistics on Accidents at Work (ESAW)*.

[CIT0075] Fino P., Lockhart T. E. (2014). Required Coefficient of Friction during Turning at Self-selected Slow, Normal, and Fast Walking Speeds. *Journal of Biomechanics*.

[CIT0076] Fino P. C., Lockhart T. E., Fino N. F. (2015). Corner Height Influences Center of Mass Kinematics and Path Trajectory during Turning. *Journal of Biomechanics*.

[CIT0077] Flach J. M., Carroll J. S., Dainoff M. J., Hamilton W. I. (2015). Striving for Safety: Communicating and Deciding in Sociotechnical Systems. *Ergonomics*.

[CIT0078] Fong D. T. P., Hong Y., Li J. X. (2005). Lower-extremity Gait Kinematics on Slippery Surfaces in Construction Worksites. *Medicine & Science in Sports & Exercise*.

[CIT0079] Gao C., Abeysekera J. (2004). A Systems Perspective of Slip and Fall Accidents on Icy and Snowy Surfaces. *Ergonomics*.

[CIT0080] Gauchard G., Chau N., Mur J. M., Perrin P. (2001). Falls and Working Individuals: Role of Extrinsic and Intrinsic Factors. *Ergonomics*.

[CIT0081] Gaudart C. (2000). Conditions for Maintaining Ageing Operators at Work – A Case Study Conducted at an Automobile Manufacturing Plant. *Applied Ergonomics*.

[CIT0082] Gaudez C., Leclercq S., Derosier C. (2006). National Statistics of Occupational Accidents on the Level in France.

[CIT0083] Gillespie L. D. (2013). Preventing Falls in Older People: The Story of a Cochrane Review. *Cochrane Database of Systematic Reviews*.

[CIT0084] Grabiner M. D., Koh T. J., Lundin T. M., Jahnigen D. W. (1993). Kinematics of Recovery from a Stumble. *Journal of Gerontology*.

[CIT0085] Grönqvist R. (1995). Mechanisms of Friction and Assessment of Slip Resistance of New and Used Footwear Soles on Contaminated Floors. *Ergonomics*.

[CIT0086] Grönqvist R., Abeysekera J., Gard G., Hsiang S. M., Leamon T. B., Newman D. J., Gielo-Perczak K., Lockhart T. E., Pai C. Y. C. (2001). Human-centred Approaches in Slipperiness Measurement. *Ergonomics*.

[CIT0087] Grönqvist R., Hirvonen M., Tuusa A. (1993). Slipperiness of the Shoe-floor Interface: Comparison of Objective and Subjective Assessments. *Applied Ergonomics*.

[CIT0088] Guo H. R., Tanaka S., Halperin W. E., Cameron L. L. (1999). Back Pain Prevalence in US Industry and Estimates of Lost Workdays. *American Journal of Public Health*.

[CIT0089] Hämäläinen P., Takala J., Saarela K. L. (2006). Global Estimates of Occupational Accidents. *Safety Science*.

[CIT0090] Hanson J. P., Redfern M. S., Mazumdar M. (1999). Predicting Slips and Falls Considering Required and Available Friction. *Ergonomics*.

[CIT0091] Harris G. W., Shaw S. R. (1988). Slip Resistance of Floors: Users’ Opinions, Tortus Instrument Readings and Roughness Measurement. *Journal of Occupational Accidents*.

[CIT0092] Haslam R. A., Bentley T. A. (1999). Follow-up Investigations of Slip, Trip and Fall Accidents among Postal Delivery Workers. *Safety Science*.

[CIT0093] Haslam R., Stubbs D. (2006). *Understanding and Preventing Falls*.

[CIT0094] Haywood K. M. (1986). *Life Span Motor Development*.

[CIT0095] Health and Safety Executive (2014). Slips and Trips and Falls from Height in Great Britain. *Health and Safety Executive*.

[CIT0096] Hirvonen M., Leskinen T., Grönqvist R., Saario J. (1994). Detection of near Accidents by Measurement of Horizontal Acceleration of the Trunk. *International Journal of Industrial Ergonomics*.

[CIT0097] Hsu Y. W., Li K. W. (2010). A Field Assessment of Floor Slipperiness in a Fish Market in Taiwan. *Safety Science*.

[CIT0098] Hu X., Qu X. (2013). Differentiating Slip-induced Falls from Normal Walking and Successful Recovery after Slips Using Kinematic Measures. *Ergonomics*.

[CIT0099] Huang Y. H., Chen J. C., DeArmond S., Cigularov K., Chen P. Y. (2007). Roles of Safety Climate and Shift Work on Perceived Injury Risk: A Multi-level Analysis. *Accident Analysis and Prevention*.

[CIT0100] Hunting K. L., Matanoski G. M., Larson M., Wolford R. (1991). Solvent Exposure and the Risk of Slips, Trips, and Falls among Painters. *American Journal of Industrial Medicine*.

[CIT0101] Jensen O. C., Sørensen J. F. L., Canals M. L., Hu Y., Nikolic N., Mozer A. A. (2005). Non-fatal Occupational Injuries Related to Slips, Trips and Falls in Seafaring. *American Journal of Industrial Medicine*.

[CIT0102] Joh A. S., Adolph K. E., Campbell M. R., Eppler M. A. (2006). Why Walkers Slip: Shine is Not a Reliable Cue for Slippery Ground. *Perception and Psychophysics*.

[CIT0103] Kemmlert K., Lundholm L. (1998). Slips, Trips and Falls in Different Work Groups with Reference to Age. *Safety Science*.

[CIT0104] Kim I. J. (2015). Wear Observation of Shoe Surfaces: Application for Slip and Fall Safety Assessments. *Tribology Transactions*.

[CIT0105] Kim S., Lockhart T., Yoon H. Y. (2005). Relationship between Age-related Gait Adaptations and Required Coefficient of Friction. *Safety Science*.

[CIT0106] Kines P. (2003). Case Studies of Occupational Falls from Heights: Cognition and Behavior in Context. *Journal of Safety Research*.

[CIT0107] Koepp G. A., Snedden B. J., Levine J. A. (2015). Workplace Slip, Trip and Fall Injuries and Obesity. *Ergonomics*.

[CIT0108] Kong P. W., Suyama J., Hostler D. (2013). A Review of Risk Factors of Accidental Slips, Trips, and Falls among Firefighters. *Safety Science*.

[CIT0109] Lanshammar H., Strandberg L., Matsui H., Kobayashi K. (1983). Horizontal Floor Reaction Forces and Heel Movements during the Initial Stance Phase. *Biomechanics VIII*.

[CIT0110] Leamon T. B., Li K. W. (1990). Microslip Length and the Perception of Slipping. *Proceedings of the 23rd International Advances in Industrial Congress on Occupational Health*.

[CIT0111] Leamon T. B., Murphy P. L. (1995). Occupational Slips and Falls: More than a Trivial Problem. *Ergonomics*.

[CIT0112] Leclercq S. (2002). Prevention of Falls on the Level in Occupational Situations: A Major Issue, a Risk to Be Managed. *International Journal of Occupational Safety and Ergonomics*.

[CIT0113] Leclercq S. (2014). Organisational Factors of Occupational Accidents with Movement Disturbance (OAMD) and Prevention. *Industrial Health*.

[CIT0114] Leclercq S., Cuny-Guerrier A., Gaudez C., Aublet-Cuvelier A. (2015). Similarities between Work Related Musculoskeletal Disorders and Slips, Trips and Falls. *Ergonomics*.

[CIT0115] Leclercq S., Monteau M., Cuny X. (2010). Avancée Dans La Prévention Des «Chutes De Plain-Pied» Au Travail. Proposition De Définition Opérationnelle D’une Nouvelle Classe: «Les Accidents Avec Perturbation Du Mouvement (APM). PISTES.

[CIT0116] Leclercq S., Saulnier H. (2002). Floor Slip Resistance Changes in Food Sector Workshops: Prevailing Role Played by “Fouling”. *Safety Science*.

[CIT0117] Leclercq S., Saurel D., Cuny X., Monteau M. (2014). Research into Cases of Slips, Collisions and Other Movement Disturbances Occurring in Work Situations in a Hospital Environment. *Safety Science*.

[CIT0118] Leclercq S., Thouy S. (2004). Systemic Analysis of So-called ‘Accidents on the Level’ in a Multi Trade Company. *Ergonomics*.

[CIT0119] Leclercq S., Thouy S., Rossignol E. (2007). Progress in Understanding Processes Underlying Occupational Accidents on the Level Based on Case Studies. *Ergonomics*.

[CIT0120] Lesch M. F., Chang W. R., Chang C. C. (2008). Visually Based Perceptions of Slipperiness: Underlying Cues, Consistency and Relationship to Coefficient of Friction. *Ergonomics*.

[CIT0121] Li K. W., Chang W. R., Leamon T. B., Chen C. J. (2004). Floor Slipperiness Measurement: Friction Coefficient, Roughness of Floors, and Subjective Perception under Spillage Conditions. *Safety Science*.

[CIT0122] Li K. Y., Chen C. J. (2005). Effects of Tread Groove Orientation and Width of the Footwear Pads on Measured Friction Coefficients. *Safety Science*.

[CIT0123] Li K. W., Hsu Y. W., Chang W. R., Lin C. H. (2007). Friction Measurements on Three Commonly Used Floors on a College Campus under Dry, Wet, and Sand-covered Conditions. *Safety Science*.

[CIT0124] Li K. W., Meng F., Zhang W. (2014). Friction between Footwear and Floor Covered with Solid Particles under Dry and Wet Conditions. *International Journal of Occupational Safety and Ergonomics*.

[CIT0125] Li K. W., Wu H. H., Lin Y. C. (2006). The Effect of Shoe Sole Tread Groove Depth on the Friction Coefficient with Different Tread Groove Widths, Floors and Contaminants. *Applied Ergonomics*.

[CIT0126] Li K. W., Yu R., Zhang W. (2011). Roughness and Slipperiness of Floor Surface: Tactile Sensation and Perception. *Safety Science*.

[CIT0127] Liberty Mutual Research Institute for Safety (2012). 2012 Workplace Safety Index. *From Research to Reality*.

[CIT0128] Liberty Mutual Research Institute for Safety (2014). 2014 Workplace Safety Index. *From Research to Reality*.

[CIT0129] Liu J., Lockhart T. E. (2006). Comparison of 3D Joint Moments Using Local and Global Inverse Dynamics Approaches among Three Different Age Groups. *Gait & Posture*.

[CIT0130] Liu J., Lockhart T. E., Kim S. (2014). Reaction Moment at the L5/S1 Joint during Simulated Forward Slipping with a Handheld Load. *International Journal of Occupational Safety and Ergonomics*.

[CIT0131] Liu J., Lockhart T. E., Parijat P., McIntosh J. D., Chiu Y. P. (2015). Comparison of Slip Training in VR Environment and on Movement Platform. *Biomedical Sciences Instrumentation*.

[CIT0132] Lockhart T. E. (1997). The Ability of Elderly People to Traverse Slippery Walking Surfaces.

[CIT0133] Lockhart T. E. (2008). An Integrated Approach towards Identifying Age-related Mechanisms of Slip Initiated Falls. *Journal of Electromyography and Kinesiology*.

[CIT0134] Lockhart T. E. (2010). Kinetic Learning in Occupational Fall Prevention Training.

[CIT0135] Lockhart T. E., Liu J. (2008). Differentiating Fall-prone and Healthy Adults Using Local Dynamic Stability. *Ergonomics*.

[CIT0136] Lockhart T. E., Smith J. L., Woldstad J. C. (2005). Effects of Aging on the Biomechanics of Slips and Falls. *Human Factors: The Journal of the Human Factors and Ergonomics Society*.

[CIT0137] Lockhart T. E., Spaulding J., Park S. H. (2007). Age-related Slip Avoidance Strategy While Walking over a Known Slippery Floor Surface. *Gait and Posture*.

[CIT0138] Lockhart T. E., Woldstad J. C., Smith J. L. (2003). Effects of Age-related Gait Changes on the Biomechanics of Slips and Falls. *Ergonomics*.

[CIT0139] Lortie M., Rizzo P. (1999). Reporting and Classification of Loss of Balance Accidents. *Safety Science*.

[CIT0140] Manning D. P., Jones C. (2001). The Effect of Roughness, Floor Polish, Water, Oil and Ice on Underfoot Friction:: Current Safety Footwear Solings are Less Slip Resistant than Microcellular Polyurethane. *Applied Ergonomics*.

[CIT0141] Manning D. P., Mitchell R. G., Blanchfield L P (1984). Body Movements and Events Contributing to Accidental and Nonaccidental Back Injuries. *Spine*.

[CIT0142] Manning D. P., Shannon H. S. (1981). Slipping Accidents Causing Low-Back Pain in a Gearbox Factory. *Spine*.

[CIT0143] Mattila M. S., Grönqvist R. A., Lankinen T. T., Leskinen T. P. J., Plaketti P. J., Ikonen K. J., Suvensalmi J. A. K. (2006). Safer Work for Electricians in Hazardous Conditions.

[CIT0144] Maynard W. S., Robertson M. M. (2007). Application of Tribology Research: Prevention of Slips, Trips and Falls.

[CIT0145] Mearns K., Flin R. (1996). Risk Perceptions in Hazardous Industries. *Psychologist*.

[CIT0146] Menant J. C., Steele J. R., Menz H. B., Munro B. J., Lord S. R. (2009). Effects of Walking Surfaces and Footwear on Temporo-spatial Gait Parameters in Young and Older People. *Gait and Posture*.

[CIT0147] Miller E. M., Matrangola S. L., Madigan M. L. (2011). Effects of Obesity on Balance Recovery from Small Postural Perturbations. *Ergonomics*.

[CIT0148] Mills R., Dwyer-Joyce R. S., Loo-Morrey M. (2009). The Mechanisms of Pedestrian Slip on Flooring Contaminated with Solid Particles. *Tribology International*.

[CIT0149] Mohan R., Das B. N., Sundaresan R. (2015). Effect of Hardness and Surface Roughness on Slip Resistance of Rubber. *Journal of Testing and Evaluation*.

[CIT0150] Moghaddam S. R. M., Redfern M. S., Beschorner K. E. (2015). A Microscopic Finite Element Model of Shoe-floor Hysteresis and Adhesion Friction. *Tribology Letters*.

[CIT0151] Moyer B. E., Chambers A. J., Redfern M. S., Cham R. (2006). Gait Parameters as Predictors of Slip Severity in Younger and Older Adults. *Ergonomics*.

[CIT0152] Myung R., Smith J. L., Leamon T. B. (1993). Subjective Assessment of Floor Slipperiness. *International Journal of Industrial Ergonomics*.

[CIT0153] Nagata H. (1989). The Methodology of Insuring the Validity of a Slip-resistance Meter.

[CIT0154] Nenonen N. (2013). Analysing Factors Related to Slipping, Stumbling, and Falling Accidents at Work: Application of Data Mining Methods to Finnish Occupational Accidents and Diseases Statistics Database. *Applied Ergonomics*.

[CIT0155] Nicholson A. S., David G. C. (1985). Slipping, Tripping and Falling Accidents to Delivery Drivers. *Ergonomics*.

[CIT0156] Owings T. M., Pavol M. J., Grabiner M. D. (2001). Mechanisms of Failed Recovery following Postural Perturbations on a Motorized Treadmill Mimic Those Associated with an Actual Forward Trip. *Clinical Biomechanics*.

[CIT0157] Parijat P., Lockhart T. E., Liu J. (2015a). EMG and Kinematic Responses to Unexpected Slips after Slip Training in Virtual Reality. *IEEE Transactions on Biomedical Engineering*.

[CIT0158] Parijat P., Lockhart T. E., Liu J. (2015b). Effects of Perturbation-based Slip Training Using a Virtual Reality Environment on Slip-induced Falls. *Annals of Biomedical Engineering*.

[CIT0159] Pater R. (1985). How to Reduce Falling Injuries. *National Safety and Health News*.

[CIT0160] Patla A. E., Patla A. E. (1991). Visual Control of Human Locomotion. *Adaptability of Human Gait: Implications for the Control of Locomotion*.

[CIT0161] Perkins P. J., Anderson C., Senne J. (1978). Measurements of Slip between the Shoe and Ground during Walking. *American Society of Testing and Materials: Special Technical Publication*.

[CIT0162] Perkins P. J., Wilson M. P. (1983). Slip Resistance Testing of Shoes – New Developments. *Ergonomics*.

[CIT0163] Perrin P., Lestienne F. (1994). *Mécanismes De L’Equilibration Humaine*.

[CIT0164] Perry J. (1992). *Gait Analysis: Normal and Pathological Function*.

[CIT0165] Pijnappels M., Bobbert M. F., van Dieën J. H. (2005a). How Early Reactions in the Support Limb Contribute to Balance Recovery after Tripping. *Journal of Biomechanics*.

[CIT0166] Pijnappels M., Bobbert M. F., van Dieën J. H. (2005b). Control of Support Limb Muscles in Recovery after Tripping in Young and Older Subjects. *Experimental Brain Research*.

[CIT0167] Pijnappels M., Bobbert M. F., van Dieën J. H. (2005c). Push-off Reactions in Recovery after Tripping Discriminate Young Subjects, Older Non-fallers and Older Fallers. *Gait and Posture*.

[CIT0168] Pyykkö I., Jantti P., Aalto H. (1990). Postural Control in Elderly Subjects. *Age and Ageing*.

[CIT0169] Quirion F., Poirier P., Lehane P. (2008). Improving the Cleaning Procedure to Make Kitchen Floors Less Slippery. *Ergonomics*.

[CIT0170] Redfern M. S., Schumann T. (1994). A Model of Foot Placement during Gait. *Journal of Biomechanics*.

[CIT0171] Redfern M. S., Cham R., Gielo-Perczak K., Grönqvist R., Hirvonen M., Lanshammar H., Marpet M., Pai C. Y. C., Powers C. (2001). Biomechanics of Slips. *Ergonomics*.

[CIT0172] Reed-Jones J. G., Reed-Jones R. J., Hollands M. A. (2014). Is the Size of the Useful Field of View Affected by Postural Demands Associated with Standing and Stepping?. *Neuroscience Letters*.

[CIT0173] Rich B. M. (2012). Integrating Safety with Science, Technology and Innovation at Los Alamos National Laboratory. http://permalink.lanl.gov/object/tr?what=info:lanl-repo/lareport/LA-UR-12-20335.

[CIT0174] Riihimäki H. (1985). Back Pain and Heavy Physical Work: A Comparative Study of Concrete Reinforcement Workers and Maintenance House Painters. *British Journal of Industrial Medicine*.

[CIT0175] Rohrlich J. T., Sadhu A., Sebastian A., Ahn N. (2014). Risk Factors for Nonorganic Low Back Pain in Patients with Worker's Compensation. *The Spine Journal*.

[CIT0176] Rowland F. J., Jones C., Manning D. P. (1996). Surface Roughness of Footwear Soling Materials: Relevance to Slip-resistance. *Journal of Testing and Evaluation*.

[CIT0177] van Schooten K. S., Rispens S. M., Pijnappels M., Daffertshofer A., van Dieen J. H. (2013). Assessing Gait Stability: The Influence of State Space Reconstruction on Inter- and Intra-day Reliability of Local Dynamic Stability during Over-ground Walking. *Journal of Biomechanics*.

[CIT0178] Schulz B. W. (2011). Minimum Toe Clearance Adaptations to Floor Surface Irregularity and Gait Speed. *Journal of Biomechanics*.

[CIT0179] Sicre A., Leclercq S., Gaudez C., Gauthier G., Vercher J., Bourdin C. (2008). Modelling Gait Processes as a Combination of Sensory-motor and Cognitive Controls in an Attempt to Describe Accidents on the Level in Occupational Situations. *Industrial Health*.

[CIT0180] Singh G., Beschorner K. E. (2014). A Method for Measuring Fluid Pressures in the Shoe-Floor-Fluid Interface: Application to Shoe Tread Evaluation. *IIE Transactions on Occupational Ergonomics and Human Factors*.

[CIT0181] Staal C., White B., Brasser B., LeForge L., Dlouhy A., Gabier J. (2004). Reducing Employee Slips, Trips and Falls during Employee-assisted Patient Activities. *Rehabilitation Nursing*.

[CIT0182] Stergiou N. (2004). *Innovative Analyses of Human Movement*.

[CIT0183] Strandberg L., Lanshammar H. (1981). The Dynamics of Slipping Accidents. *Journal of Occupational Accidents*.

[CIT0184] Strandberg L. (1983). On Accident Analysis and Slip-resistance Measurement. *Ergonomics*.

[CIT0185] Strandberg L. (1985). The Effect of Conditions Underfoot on Falling and Overexertion Accidents. *Ergonomics*.

[CIT0186] Swedler D. I., Verma S. K., Huang Y.-H., Lombardi D. A., Chang W. R., Brennan M., Courtney T. K. (2015). A Structural Equation Modelling Approach Examining the Pathways between Safety Climate, Behaviour Performance and Workplace Slipping. *Occupational and Environmental Medicine*.

[CIT0187] Swensen E. E., Purswell J. L., Schlegel R. E. (1992). Coefficient of Friction and Subjective Assessment of Slippery Work Surfaces. *Human Factors: The Journal of the Human Factors and Ergonomics Society*.

[CIT0188] Tisserand M. (1969). Criteres D’adherences Des Semelles De Securite.

[CIT0189] Tisserand M. (1985). Progress in the Prevention of Falls Caused by Slipping. *Ergonomics*.

[CIT0190] Underwood D. C. (1991). Effect of Floor Soil on Coefficient of Friction in Foodservice Operations. *Ceramica Acta*.

[CIT0191] Verma S. K., Chang W. R., Courtney T. K., Lombardi D. A., Huang Y. H., Brennan M. J., Mittleman M. A., Perry M. J. (2010). Workers’ Experience of Slipping in US Limited Service Restaurants. *Journal of Occupational & Environmental Hygiene*.

[CIT0192] Verma S. K., Chang W. R., Courtney T. K., Lombardi D. A., Huang Y. H., Brennan M. J., Mittleman M. A., Ware J. H., Perry M. J. (2011). A Prospective Study of Floor Surface, Shoes, Floor Cleaning and Slipping in US Limited-service Restaurant Workers. *Occupational Environmental Medicine*.

[CIT0193] Videman T., Nurminen T., Tola S., Kuorinka I., Vanharanta H., Troup J. D. G. (1984). Low-back Pain in Nurses and Some Loading Factors of Work. *Spine*.

[CIT0194] Wilson M. P., Gray B. E. (1990). Development of SATRA Slip Test and Tread Pattern Design Guidelines. *Slips, Stumbles, and Falls: Pedestrian Footwear and Surfaces*.

[CIT0195] Wu X., Lockhart T. E., Yeoh H. (2012). Effects of Obesity on Slip-induced Fall Risks among Young Male Adults. *Journal of Biomechanics*.

[CIT0196] Yamaguchi T., Yano M., Onodera H., Hokkirigawa K. (2013). Kinematics of Center of Mass and Center of Pressure Predict Friction Requirement at Shoe–Floor Interface during Walking. *Gait and Posture*.

[CIT0197] Yeoh H. T., Lockart T. E., Wu X. (2013). Non-fatal Occupational Falls on the Same Level. *Ergonomics*.

[CIT0198] Yoshioka M., Ono H., Kawamura S., Miyaki M. (1978). On Slipperiness of Building Floors – Fundamental Investigation for Scaling of Slipperiness.

[CIT0199] Yoshioka M., Ono H., Shinohara M., Kawamura S., Miyaki M., Kawata A. (1979). Slipperiness of Building Floors. *Report of the Research Laboratory of Engineering Materials, No. 4*.

[CIT0200] Zhang J., Lockhart T. E., Soangra R. (2015). Classifying Lower Extremity Muscle Fatigue during Walking Using Machine Learning and Inertial Sensors. *Annals of Biomedical Engineering*.

[CIT0201] Zohar D. (1978). Why Do We Bump into Things While Walking?. *Human Factors: The Journal of the Human Factors and Ergonomics Society*.

[CIT0202] Zohar D., Quick J. C., Tetrick L. E. (2003). Safety Climate: Conceptual and Measurement Issues. *Handbook of Occupational Health Psychology*.

[CIT0203] Zohar D. (2010). Thirty Years of Safety Climate Research: Reflections and Future Directions. *Accident Analysis and Prevention*.

